# Rapid restructuring of the odontocete community in an ocean warming hotspot

**DOI:** 10.1111/gcb.16382

**Published:** 2022-08-23

**Authors:** Lesley H. Thorne, Eleanor I. Heywood, Nathan O. Hirtle

**Affiliations:** ^1^ School of Marine and Atmospheric Sciences Stony Brook University Stony Brook New York USA

**Keywords:** cetacean, climate response, distributional shift, rapid warming, regional change, trailing edge

## Abstract

Cetaceans are important consumers in marine ecosystems, but few studies have quantified their climate responses. The rapid, directional warming occurring in the Northeast United States (NEUS) provides a unique opportunity to assess climate impacts on cetaceans. We used stranding data to examine changes to the distribution and relative abundance of odontocetes from 1996 to 2020 in both the NEUS and the Southeast United States (SEUS), which is not warming. We conducted simulations to determine the number of stranding events needed to detect a distributional shift for each species given the speed of the shift and the spatial variability in strandings. We compared observed shifts to climate velocity. Smaller sample sizes were needed to detect more rapid poleward shifts, particularly for species with low spatial variability. Poleward shifts were observed in all species with sufficient sample sizes, and shifts were faster than predicted by climate velocity. For species whose trailing edge of distribution occurred in the NEUS, the center of distribution approached the northern limit of the NEUS and relative abundance declined through time, suggesting shifts north out of US waters. The relative abundance of warm water species in the stranding record increased significantly in the NEUS while that of cool water species declined significantly as their distributions shifted north out of the NEUS. Changes in the odontocete community were less apparent in the SEUS, highlighting the importance of regional warming. Observed poleward shifts and changes in species composition suggest a reorganization of the odontocete community in the NEUS in response to rapid warming. We suggest that strandings provide a key dataset for understanding climate impacts on cetaceans given limitations of survey effort and modeling approaches for predicting distributions under rapidly changing conditions. Our findings portend marked changes to the distribution of highly mobile consumer species across international boundaries under continued warming.

## Graphical abstract

Odontocetes are important consumers in marine ecosystems, but few studies have quantified their climate responses. Waters of the Northeast United States (NEUS) are undergoing rapid, directional warming. Using stranding data, we observed poleward shifts in the distribution of strandings for all odontocete species with sufficient data. We found that the relative abundance of warm water odontocete species in the stranding record increased significantly in the NEUS while that of cool water species declined significantly as their distributions shifted north out of US waters. Our results suggest a reorganization of the odontocete community in the NEUS in response to rapid warming.

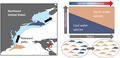

## 1 INTRODUCTION

Climate‐driven distributional changes have been observed across a wide range of marine species and can result in fundamental changes to community structure and ecosystem processes (Doney et al., 2012; Pecl et al., 2017; Poloczanska et al., 2013; Wernberg et al., 2016). The velocity of climate change is higher in marine systems than in terrestrial systems, and marine species generally shift faster than terrestrial species (Burrows et al., 2011; Pinsky et al., 2020; Poloczanska et al., 2013; Sorte et al., 2010). Cetaceans are large, long‐lived marine predators with key ecological functions and are often considered to be sentinels of changes to marine ecosystems and food webs (Aguirre & Tabor, 2004; Hazen et al., 2019; Moore, 2008), but the impacts of climate change on cetaceans are not well understood (Poloczanska et al., 2016; Silber et al., 2017). Many studies have suggested how climate change might influence cetaceans and other marine mammals, but due to insufficient research and monitoring focused on assessing climate impacts, few have demonstrated links (Gulland et al., 2022; Silber et al., 2017; van Weelden et al., 2021). For example, warming waters associated with climate change are expected to have marked impacts on the distribution and life history of cetaceans (Chambault et al., 2018; MacLeod, 2009; Wild et al., 2019), but range shifts in these species are seldom quantified (Poloczanska et al., 2016; Thorne & Nye, 2021). Understanding distributional shifts and changes to marine mammal community composition is key to developing effective monitoring and management strategies for these species, and to understanding impacts of distributional changes on marine food webs (Albouy et al., 2020; MacLeod, 2009; Thorne & Nye, 2021).

Long‐term studies are needed to assess climate responses of cetaceans, but such studies are challenging as cetaceans are highly mobile species that occur at low densities and spend most of their lives underwater. Line‐transect surveys provide key information on cetacean abundance and distribution (Hammond et al., 2013), but limited data and variability in survey coverage hamper efforts to detect trends in cetacean abundance and distribution at large spatial scales (Jewell et al., 2012; Kaschner et al., 2012). Species distribution models (SDMs) developed using data from line transect surveys allow distributions to be forecasted over different time periods (Becker et al., 2012, 2019; Bouchet et al., 2019; Thorne et al., 2019), but predictions from SDMs may be less accurate over long time frames or in times and places of rapid warming (Araújo et al., 2005; Franklin, 2010; Jeschke & Strayer, 2008; Zurell et al., 2009). Strandings data can provide an alternative data source for examining trends in the occurrence, distribution, and species composition of cetaceans (MacLeod, 2009; Pyenson, 2010, 2011; Thorne & Nye, 2021). Databases of stranding events have been maintained for decades in some countries, providing a valuable record for analyses of strandings relative to oceanographic and climatic change (Pyenson, 2011). When using stranding records to assess distributional shifts, it is important to consider sample sizes needed to detect trends as the abundance of different cetacean species, as well as their representation and spatial distribution in the stranding record varies considerably. Furthermore, rapid shifts may be detected using fewer data than slower shifts, and thus the pace of distributional change is another important consideration when assessing data requirements for quantifying changes to distributions through time. Simulation and modeling approaches can provide valuable means of quantifying the level of confidence in range shifts of marine species, particularly given variability in sample size (Bates et al., 2015).

Recent studies have shown some disagreement as to whether marine species show faster climate responses at the “trailing” (equatorward) or “leading” (poleward) edges of their distributions (Poloczanska et al., 2013; Sunday et al., 2015). Some studies have demonstrated faster changes occur at trailing edges (Robinson et al., 2015), while others have observed faster changes at leading edges (Fredston‐Hermann et al., 2020; Poloczanska et al., 2013), or similar changes at both trailing and leading edges (Sunday et al., 2012). Assessing distributional shifts at both the center and edges of distributions would provide an improved understanding of species' range expansions and contractions, and implications for habitat loss and management of cetacean species under continued warming.

Ocean warming hotspots can provide natural laboratories for understanding the consequences and challenges of climate change, and can thus serve as early warning systems of future impacts (Pecl et al., 2014). Temperate coastal systems such as the Northeast United States (NEUS) have experienced dramatic oceanographic and ecological change in recent decades. Water temperatures in the NEUS have experienced some of the highest rates of warming globally in recent decades (Pershing et al., 2015). Climate‐driven poleward shifts have been observed in the distribution of several species of fish and invertebrates in association with this warming (Murawski, 1993; Nye et al., 2009; Pinsky et al., 2013). Shifts in the summer foraging areas of baleen whales in the NEUS have been associated with climate‐driven oceanographic change (Davies et al., 2019; Meyer‐Gutbrod et al., 2018; Simard et al., 2019) and phenological shifts in baleen whale habitat use in this region have been observed in association with changes in thermal spring transition dates (Pendleton et al., 2022). Thorne and Nye (2021) found that the distribution of long‐finned pilot whales (*Globicephala melas*) in the NEUS shifted poleward over a 25‐year period. Continuous poleward distributional shifts and rates of such shifts in the distribution of other odontocete species have not been quantified to date or linked with the pace of climate‐driven oceanographic change. In addition, the rapid warming occurring in the NEUS is not observed in the Southeast United States (SEUS), where temperature trends are not significant or weakly cooling (Shearman & Lentz, 2010). The impacts of these divergent temperature trends on species distributions are not clear. The differences in warming trends along the coastline of the eastern United States, which covers a range of approximately 20° latitude, present a unique opportunity to assess how regional warming impacts community structure and distributional shifts in cetaceans.

Here we analyze cetacean stranding records over a 25‐year period to assess how warming waters in the NEUS impacted the distribution of odontocete strandings. Odontocetes present advantages as focal species for assessing distributional shifts of cetaceans through time. While quantifying shifts in baleen whales is complicated by their extensive seasonal migrations from tropical to high‐latitude waters, odontocete species are typically resident or weakly migratory and generally have larger populations sizes. MacLeod (2009) suggested that cetacean species with a range that is limited to non‐tropical waters are more likely to be impacted by changes to water temperature. For these reasons, and because odontocetes are well represented in stranding data, we focus on odontocete species in our analyses. Our specific objectives were to (1) characterize climate velocity and regional warming along the east coast of the United States; (2) examine and compare changes in odontocete species composition in the NEUS and SEUS from stranding data; (3) assess the number of observations needed to detect distributional shifts relative to the rate of change; and (4) quantify changes in the distribution and relative abundance of strandings of odontocete species through time.

## 2 METHODS

### 2.1 Stranding data

A variety of factors have been assessed as possible drivers of stranding events, including disease, malnutrition, disorientation, military sonar, vessel strikes, and fisheries bycatch (Bogomolni et al., 2010; Geraci & Lounsbury, 2005; Jepson et al., 2003; Leeney et al., 2008; Vanselow et al., 2018). However, in many cases, the drivers of strandings are unclear. Despite the multitude of causes behind strandings, stranding records have been found to represent patterns of relative abundance and diversity in living cetacean communities (Pyenson, 2011). Here we use the location of odontocete strandings occurring throughout the eastern seaboard of the United States to examine patterns of odontocete distribution through time.

In the United States, basic information on marine mammal strandings, known as Level A data, must be collected by stranding responders and is standardized and collated by the National Marine Fisheries Service (NMFS). Level A data include information such as the species identification, location, and condition of the stranded animal. In the SEUS, stranding data were curated by NMFS as part of the Marine Mammal Health and Stranding Response Program (MMHSRP) National Database from 1996 onward (Litz et al., 2014). To ensure consistency in stranding reports and records, we, therefore, use data from the MMHSRP National Database, which includes data from both the NEUS and SEUS, and assessed changes in the distribution of strandings from 1996 to 2020. A list of all odontocete species observed in the stranding record along the eastern seaboard of the United States is provided in Table S1.

In addition to latitude and longitude, Level A stranding data detail geographic descriptions such as well as the city, county, state, and body of water. Cross‐checks with geographic descriptions revealed that some recorded stranding location coordinates or notations were inconsistent, particularly between data from the NEUS and SEUS, or were recorded incorrectly in the database. We therefore developed a protocol to correctly identify the true coordinate notation, accurately convert all coordinates to decimal degrees, and cross‐check the accuracy of stranding locations using positions geocoded from the geographic description data; this is described in Supplementary Information along with further details of data processing for stranding events.

The processed stranding dataset included 7320 stranding events in the NEUS and 7236 in the SEUS (14,556 stranding events total) from 1996 to 2020 that were used in subsequent analyses (sample size by species is shown in Table S1).

### 2.2 Framework for assessing changes in species composition

We used the framework of MacLeod (2009) to group odontocetes into four climatic categories, warm water, cool water, Arctic/ sub‐Arctic and cosmopolitan. Climatic categories for each odontocete species observed in the stranding record, taken from MacLeod (2009), are shown in Table S1. We compare how the proportion of strandings in these four categories has changed through time in the SEUS and NEUS, respectively.

We used the locations of species sightings from stock assessment reports (Hayes et al., 2021) in combination with IUCN range maps (Figure S1) and the spatial location of stranding data to identify any odontocete species for which the trailing or leading edge of their distribution occurred within the NEUS or SEUS. Of the species that were well represented within the stranding data (described below), only harbor porpoises (*Phocoena phocoena*), long‐finned pilot whales and Atlantic white‐sided dolphins (*Lagenorhynchus acutus*) showed trailing edge distributions in this region. For these species, we assessed shifts in both the center and trailing edges of their distributions. No species that were well represented in the stranding data showed leading edge distributions in the study area.

### 2.3 Climate velocity and sea surface temperature trends

**FIGURE 1:**
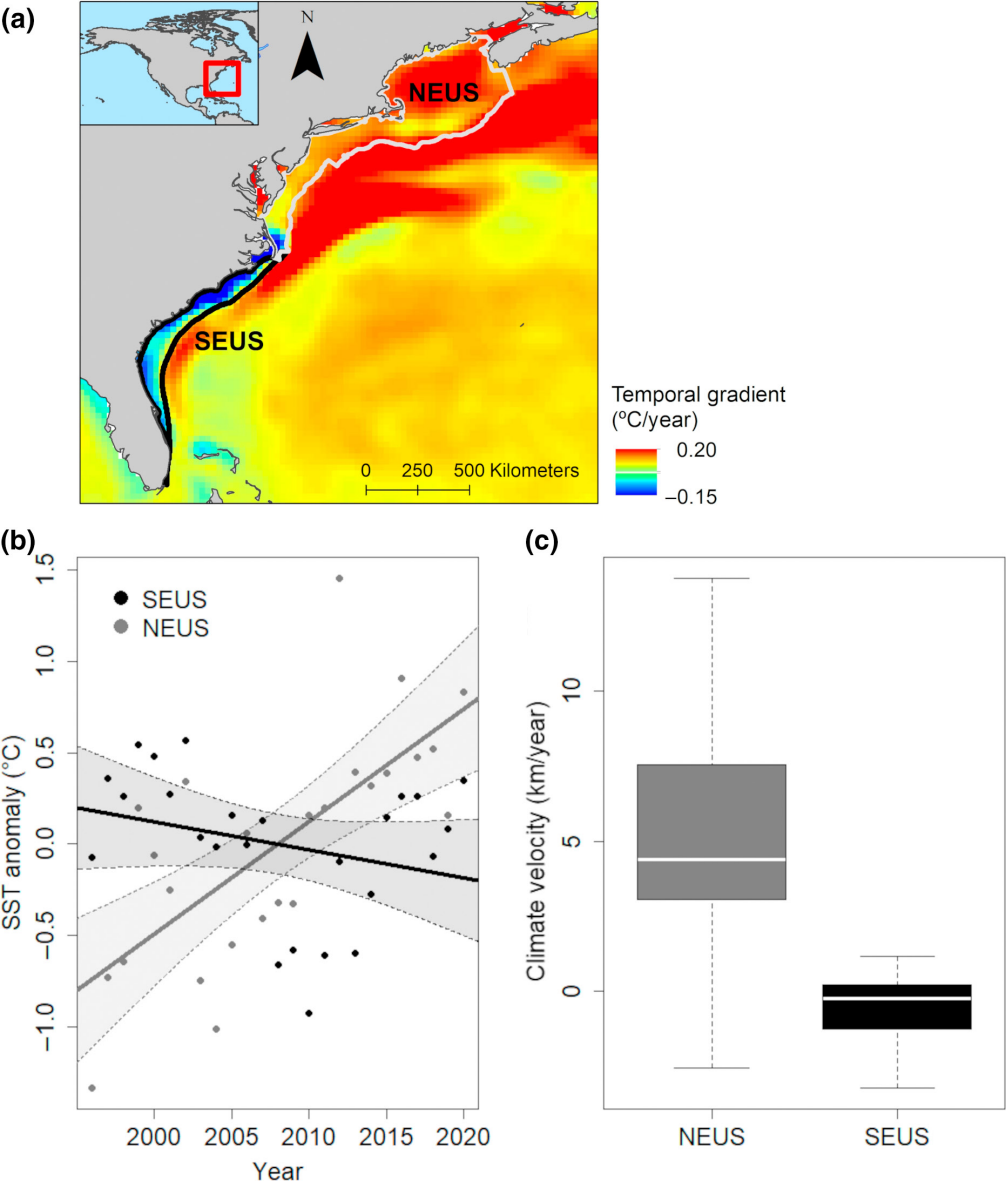
(a) Temporal gradient (°C year^−1^) in sea surface temperature (SST) in the Northwest Atlantic from 1996 to 2020. (b) Annual trends in mean SST anomaly in the northeast and Southeast United States, respectively (NEUS and SEUS) from 1996 to 2020. (c) Climate velocity in NEUS and SEUS from 1996 to 2020.

We used annual mean NOAA OI sea surface temperature (SST) data with a spatial resolution of 0.25° from 1996 to 2020, provided by the NOAA/OAR/ESRL PSL, Boulder, Colorado, USA: https://psl.noaa.gov/data/gridded/data.noaa.oisst.v2.highres.html. We used this dataset to compare annual trends in mean SST and mean annual SST anomaly between waters of the continental shelf in the NEUS and SEUS (Figure 1a) and to calculate climate velocity. We use the method proposed by Loarie et al. (2009) to assess climate velocity, which assesses the instantaneous local velocity needed to maintain a constant temperature. This approach is advantageous for ecological studies as it provides a means of assessing the pace of climate change independent of species distribution while accounting for both spatial and temporal gradients in temperature. Briefly, the ratio of temporal temperature gradients (°C year^−1^) to spatial temporal gradients (°C km^−1^) is calculated to develop an index of climate velocity in km year^−1^ (°C year^−1^/°C km^−1^ = km year^−1^). We used the VoCC packages described in Burrows et al. (2011) to calculate spatial gradients in annual SST (°C km^−1^) in a 3 × 3 cell neighborhood. The temporal gradient was calculated as the slope of the linear model of changes to SST through time (°C year^−1^).

### 2.4 Modeling temporal trends in SST, species composition, and species distributions

Following preliminary analyses indicating linear relationships between variables, we used linear regressions to assess changes in SST anomaly in the NEUS and SEUS, respectively, as well as changes in species composition and shifts in species distributions through time. We examined changes in the relative abundance of stranding for individual species, and species in each of the four climatic categories (cosmopolitan, Arctic/sub‐Arctic, cool water and warm water), and modeled these changes relative to annual SST anomaly using linear regressions. The Dunn–Šidák method was used to constrain the Type I error rate across multiple comparisons (Kirk, 1982).

For all species with an average of at least five stranding events per year (sample size requirements assessed in more detail using simulations, as below), we assessed poleward shifts in distributions. The following nine species met this criteria: harbor porpoise, bottlenose dolphins (*Tursiops truncatus*), common dolphins (*Delphinus delphis*), Atlantic white‐sided dolphins, striped dolphins (*Stenella coeruleoalba*), Risso's dolphins (*Grampus griseus*), long‐finned pilot whales, pygmy sperm whales (*Kogia breviceps*), and dwarf sperm whales (*K. sima*). Range maps for these species are shown in Figure S1 (IUCN, 2021). To assess whether poleward shifts were occurring in these species, we calculated along‐shelf distance for each stranding event as the distance from the southern end of mainland Florida (hereafter “poleward distance”) and regressed the annual mean poleward distance of stranding events with year. We examined changes in poleward distance using along‐shelf distance rather than changes in latitude due to the curvilinear shelf of the NEUS (Friedland et al., 2019; Nye et al., 2009; Thorne & Nye, 2021) and used the Spatial Analyst package in ArcGIS (version 10.8.1), with the coastline and the shelf break as barriers, to compute along‐shelf distance from the southern end of mainland Florida. For the three trailing edge species, we also modeled shifts in the location of the trailing edge of the distribution, quantified by the fifth percentile of the poleward distance of stranding events (Fredston‐Hermann et al., 2020; Liang et al., 2018). We use the results of the simulation analysis described below to assess the minimum number of stranding events needed to detect poleward shifts considering the spatial variance in stranding record for each species and the magnitude of the shift in km year^−1^. For the six species for which simulation analyses indicated sufficient data for further analyses (harbor porpoises, bottlenose dolphins, common dolphins, Atlantic white‐sided dolphins, long‐finned pilot whales and dwarf sperm whales), we compared observed shifts in the distribution of strandings and examined changes in the proportion of strandings for that species by year. We used nonparametric bootstrap sampling methods to estimate 95% confidence intervals (CIs; Efron & Tibshirani, 1986, 1994) around the slope coefficients for each species' linear model testing the hypothesis that the center or trailing edge of the strandings distribution is undergoing a poleward shift. We generated 1000 bootstrapped samples for each species, sampling with replacement the poleward distance data and conserving the distance–year row relationships. Each bootstrapped dataset consisted of the same number of events represented in the original dataset for each species. We then calculated the annual mean poleward distance, applied the same linear regression model as was fit to the original data and extracted the slope coefficients to obtain a bootstrap sampling distribution of 1000 coefficients. The 95% CIs were calculated as the 0.025 and 0.975 quantiles of the bootstrap sampling distribution of slope coefficients and are reported in Table 1.

**TABLE 1:** Characteristics of data for nine odontocete species that were assessed using power analysis simulations

Species	Mean annual sample size	Climatic grouping	*SD* poleward distance (km)	Modeled slope	Lower CI (km year^−1^)	Upper CI (km year^−1^)
Atlantic white‐sided dolphin						
Center of distribution	24	Cool water	217	5.2	2.4	8.9
Trailing edge				26.6	12.4	33.6
Long‐finned pilot whale						
Center of distribution	8	Cool water	290	*13.1*	*6.0*	*21.9*
Trailing edge				40.6	16.0	54.1
Harbor porpoise						
Center of distribution	86	Cool water	428	14.0	11.5	17.0
Trailing edge				11.2	7.4	18.7
Common dolphin	66	Warm water	307	*8.7*	*5.1*	*12.8*
Striped dolphin	7	Warm water	516	‐	‐	‐
Risso's dolphin	7	Warm water	648	‐	‐	‐
Common bottlenose dolphin	331	Warm water	569	9.3	6.4	9.6
Pygmy sperm whale	24	Warm water	733	‐	‐	‐
Dwarf sperm whale	6	Warm water	586	27.8	9.5	39.7

*Note*: Mean annual sample size represents the mean number of stranding events per year by species from 1996 to 2020. For each species, the standard deviation (*SD*) of poleward distance was calculated as the average observed poleward distance values for each species and year across all years. Modeled slope represents the slope calculated from a linear regression of the annual location (poleward distance) of the distribution for each species, while the 95% confidence intervals (CIs) were obtained by bootstrapping the underlying poleward distance data to produce a distribution of bootstrapped slope estimates (*n* = 1000). Slope values are not shown for species which did not show a significant distributional shift. The observed poleward shifts for the center of distribution for long‐finned pilot whales and common dolphins are italicized; while significant at *α* = .05, they were not statistically significant following adjustment for multiple comparisons.

### 2.5 Simulating effects of sample size, variance, and slope on the detection of poleward distributional shifts

Data limitations may influence whether distributional shifts can be detected and characterized, particularly when using data sources such as stranding records as some odontocete species strand very rarely. Our ability to detect a poleward shift over time depends on stranding event sample size, the annual variance in the poleward distance, and the slope (change in poleward distance over time). To examine the relationship between these factors and to determine the minimum mean sample size of stranding events needed to observe a trend, we simulated poleward distances for stranding events in each year with a simple linear model and varied sample size, variance in poleward distance, and slope. Details of the modeling methods are described in the Supplementary Information.

### 2.6 Assessing human population growth as a source of bias in observer effort

Human observers must be present to record marine mammal strandings along the coastline, and changes in observer coverage or human population can influence the number of strandings reported (MacLeod et al., 2005; Pyenson, 2010; Sergeant, 1982). We used coastal county US Census Bureau data as a proxy for stranding response effort, and quantified changes in human population growth in all coastal counties along the eastern seaboard to infer discrepancies or changes in effort (stranding event detection) over time (www.census.gov). Census data are available at 10‐year intervals, and thus we assessed changes in population between 2000 and 2020 to best match the timeframe for which strandings were assessed (1996–2020). Census data provided population counts for 2000 and demographic analysis estimates for 2020.

We mapped and analyzed changes in human population (change from 2000 to 2020 as a percentage of the 2000 population) to determine whether there were any spatial patterns of population growth that might impact our results (e.g., consistent poleward shifts in odontocete distribution from strandings records that mirrored human population growth at the northern extent of the observed shifts). Specifically, we assessed whether human population change was related to poleward distance at the centroid of each coastal county using linear regressions. Regressions were performed both for the east coast as a whole, and for any species showing a poleward shift in the distribution of strandings, within the poleward distances over which the shift was observed. The Dunn–Šidák method was used to adjust α for multiple comparisons (Kirk, 1982).

All analyses were conducted using R Statistical Software (v4.2.0; R Core Team, 2021).

## 3 RESULTS

Mean SST on the continental shelf of the NEUS increased rapidly at an average rate of 0.062°C year^−1^ from 1996 to 2020, while mean SST in the SEUS showed no significant change through time (Figure 1b; Table S2). There was considerable spatial variability in the temporal gradient in SST within both the SEUS and the NEUS during this time frame (Figure 1a). The northern extent of the SEUS was dominated by negative SST gradients while little change in SST was observed further south (minimum of −0.14°C year^−1^, maximum of 0.049°C year^−1^, mean of −0.015°C year^−1^ in the SEUS). Conversely, the highest temporal gradients in SST were observed in the northern NEUS (Figure 1a; Figure S2; minimum of −0.074°C year^−1^, maximum of 0.096°C year^−1^, mean of 0.062°C year^−1^ in the NEUS). Climate velocity was significantly higher in the NEUS than the SEUS during this period, with median values of 4.41 and −0.22 km year^−1^, respectively (Figure 1c). The 25% and 75% quantiles for climate velocity were 3.08 and 7.54 for the NEUS and −1.22 and 0.22 for the SEUS, respectively.

**FIGURE 2:**
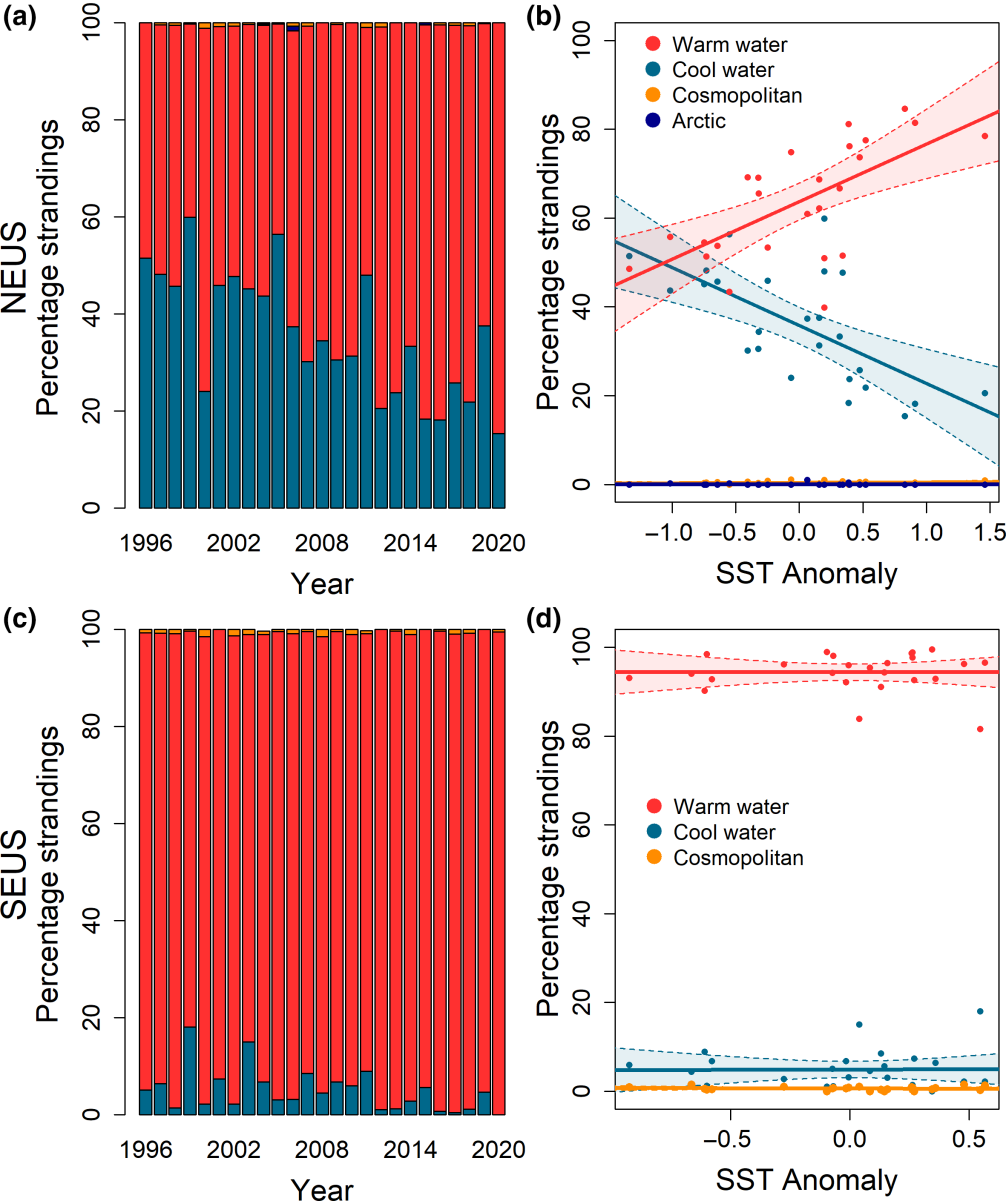
Changes in the relative abundance of odontocete strandings by climatic grouping in the Northeast United States (NEUS) and the Southeast United States (SEUS) through time (a, c) and relative to annual sea surface temperature anomaly (b, d) from 1996 to 2020. Arctic and cosmopolitan odontocete species made up less than 2% of strandings in each year in the NEUS, as did cosmopolitan species in the SEUS, and arctic species were not observed in the SEUS during the study period.

The composition of odontocete strandings in the NEUS showed marked changes through time, with a decline in the proportion of stranding events comprised of cool water species and an increase in the proportion of events comprised of warm water species over time between 1996 and 2020 (Figure 2a; Table S2). The proportion of strandings of cool water species in the NEUS decreased linearly while the proportion of strandings of warm water species increased linearly in association with SST anomaly (Figure 2b). This pattern was driven by decreases in the proportion of strandings of harbor porpoises as well as long‐finned pilot whales, and increases in the proportion of strandings of common dolphins as SST increased (Table 2; Table S2; Figure S3). This trend was particularly evident in the Gulf of Maine, at the northern extent of the NEUS, followed by Southern New England. Increases in the proportion of bottlenose dolphin strandings were evident in the southern portion of the NEUS, the Mid‐Atlantic Bight, while a decrease in the proportion of Atlantic white‐sided dolphin strandings was observed in the Gulf of Maine (Figure S4). In the SEUS, the proportion of cool water species also declined while the proportion of warm water species increased through time, though changes were not as striking as in the NEUS (no significant trend after adjustment for multiple comparisons) and showed no significant trend relative to SST anomaly in the SEUS (Figure 2c,d). In the SEUS, there was no significant change observed in the proportion of strandings comprised of any of the six focal species relative to SST anomaly (Table S2).

**TABLE 2:** Summary of observed trends for the six odontocete species that were sufficiently well represented in the strandings data to be assessed using linear models following results of simulations

Species	Poleward shift	Change in prop. strandings in NEUS	Climatic grouping
Harbor porpoise	+	−	Cool water (trailing edge)
Atlantic white‐sided dolphin	+	−	Cool water (trailing edge)
Long‐finned pilot whale	+	−	Cool water (trailing edge)
Common bottlenose dolphin	+	ND	Warm water
Common dolphin	+	+	Warm water
Dwarf sperm whale	+	ND	Warm water

*Note*: Positive trends for poleward shifts reflect northward movements through time, while changes in the proportion of strandings reflect increases or decreases in the proportion of stranding events comprised of that species in the Northeast United States (NEUS) relative to sea surface temperature anomaly. ND = no trend detected. + indicates a positive trend or a poleward shift (red) while − indicates a negative trend (blue). Common dolphins showed strong evidence of a poleward shift and 95% confidence intervals of the modeled slope of the shift did not include 0, but the shift was not statistically significant after accounting for multiple comparisons and is shown in orange. Similarly, the observed decline in the relative abundance of Atlantic white‐sided dolphins was not significant after accounting for multiple comparisons and is shown in light blue.

**FIGURE 3:**
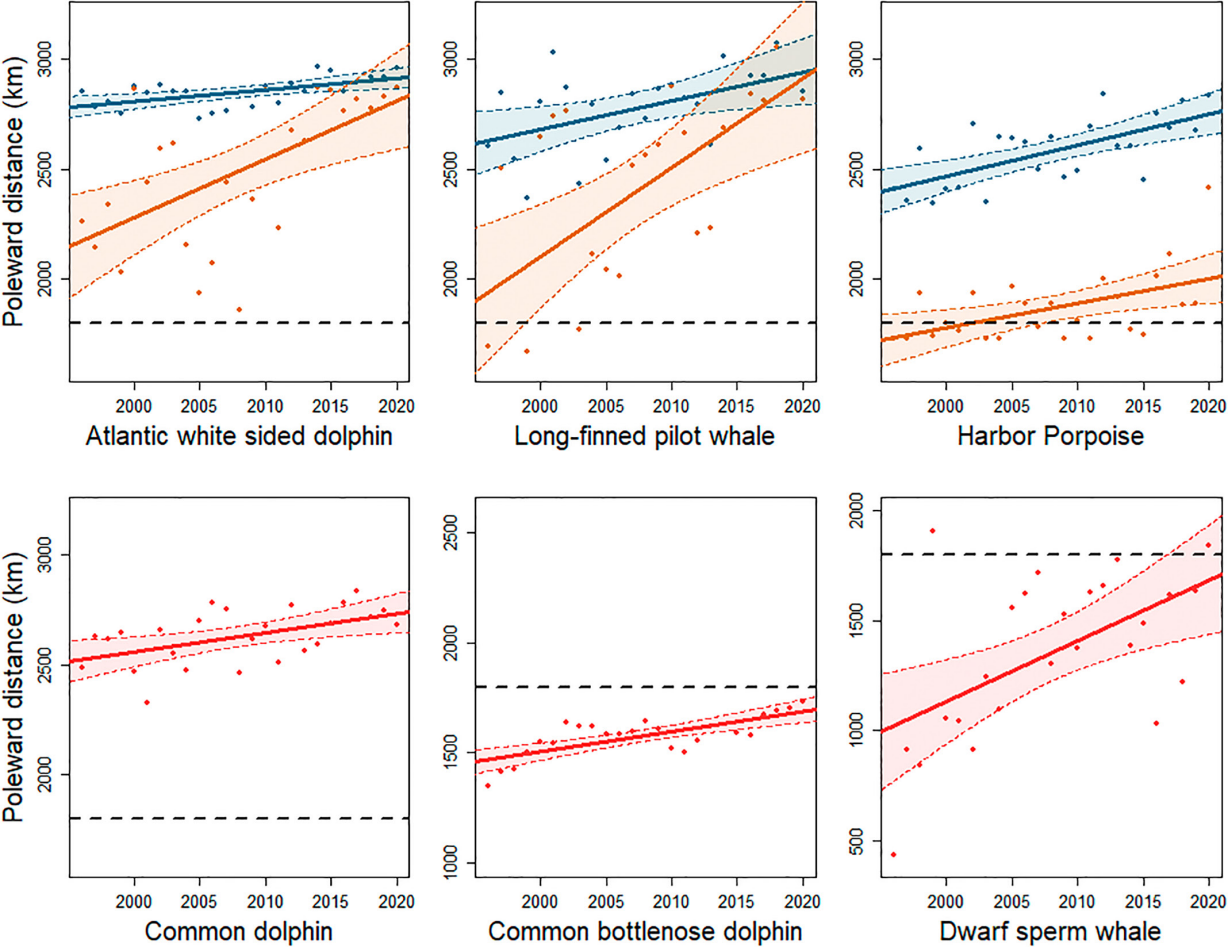
Shifts in poleward distance, measured as the along‐shelf distance from the southern tip of Florida (km), in strandings from 1996 to 2020 for six species of odontocete with sufficient data to be assessed using linear models following results of simulations. Changes in the center of distribution are shown in blue for cool water species and red for warm water species, while changes in the trailing edge of the distribution are shown in orange for the three trailing edge species. The black dotted line represents the location of Cape Hatteras, separating the Southeast and Northeast United States.

**FIGURE 4:**
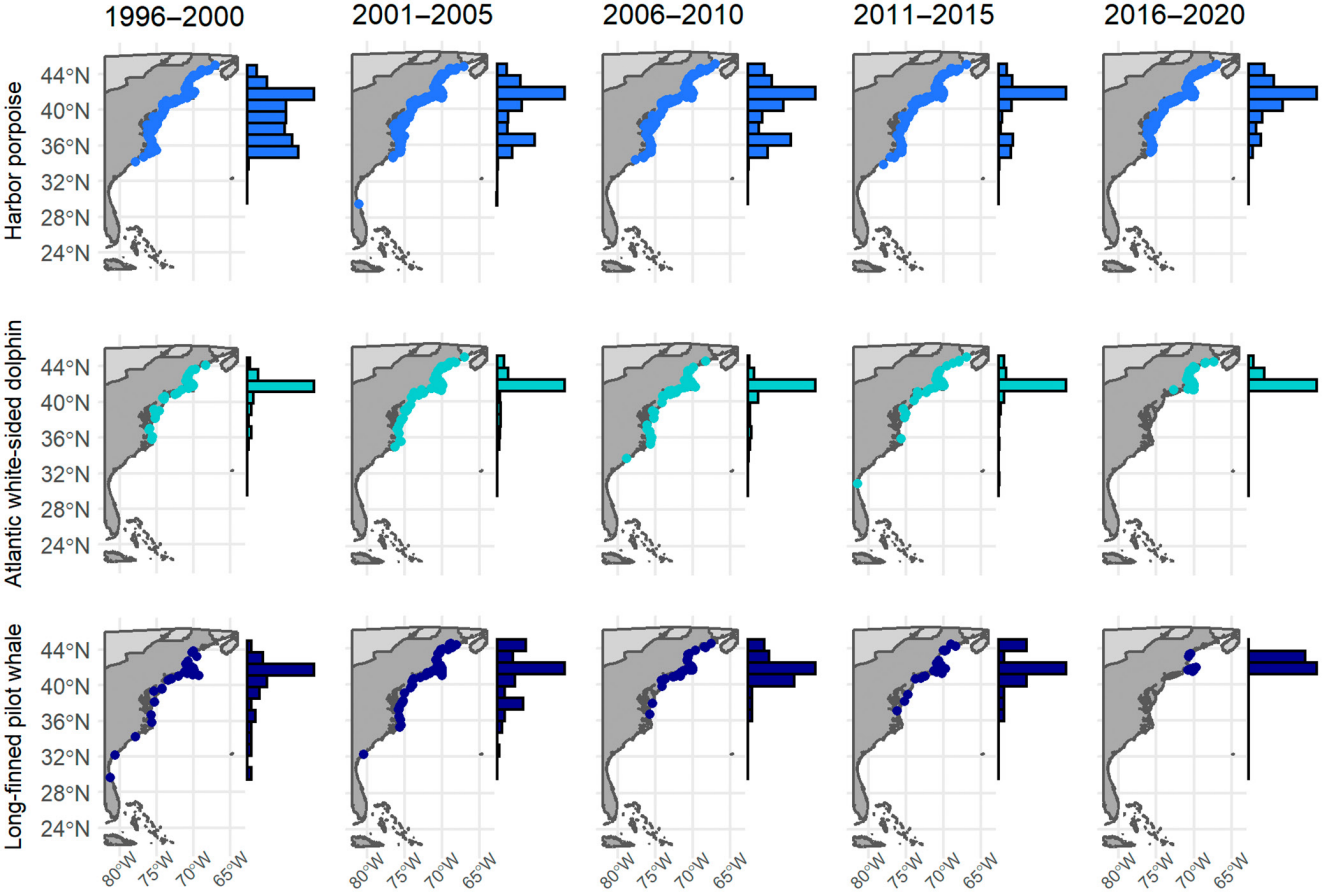
Changes in the spatial distribution of the stranding events of three cool water odontocete species, harbor porpoise, Atlantic white‐sided dolphin and long‐finned pilot whale, in 5‐year periods along the eastern seaboard of the United States. Histograms reflect the proportion of stranding events for that species in each time period binned based on latitude.

Simulation analyses indicated that available data for the three remaining warm water species (Risso's dolphins, striped dolphins and pygmy sperm whales) were insufficient to model poleward shifts (Table 3). We observed significant poleward shifts in the distribution of strandings for all three cool water species; while shifts in the trailing edge of all three species were significant, the shift in the center of distribution for long‐finned pilot whales was not significant following adjustment for multiple comparisons (Figure 3; Table 1; Table S2). Observed shifts in stranding events suggest that the distribution of long‐finned pilot whales and Atlantic white‐sided dolphins have shifted poleward close to the northern extent of the NEUS in recent years (Figures 3 and 4). Furthermore, estimates of the center of distribution and the trailing edge of the distribution for these species converged or nearly converged in recent years. Shifts in the trailing edge of these species were considerably larger than shifts in the center of distribution, with values of 26.6 km year^−1^ (95% CI 12.4–33.6 km year^−1^) for Atlantic white‐sided dolphins and 40.6 km year^−1^ (95% CI 16.0–54.1 km year^−1^) for long‐finned pilot whales, respectively (Table 1; Table S2). Two of the three warm water species with sufficient data to model poleward shifts, bottlenose dolphins and dwarf sperm whales, showed significant poleward shifts in distribution. Common dolphins showed evidence of a poleward shift and 95% CIs of the modeled slope did not include 0 (Figure 3; Figure S5; Table 1), but the observed shift was not statistically significant following adjustment for multiple comparisons (Table S2). Slopes for the center of distribution for these species ranged from 8.7 to 27.8 km year^−1^ (Table 1; Table S2). All observed shifts in odontocete species were in the poleward direction.

**TABLE 3:** Mean annual sample size (number of stranding events per year) needed to detect a significant poleward shift 95% of the time by species for slopes of −2 to 40 km year^−1^ from power analysis simulations for each species

Slope (km year^−1^)	Mean annual sample size needed to detect significant poleward shift											
	Harbor porpoise trailing edge	Harbor porpoise	Atlantic white‐sided dolphin trailing edge	Atlantic white‐sided dolphin	Long‐finned pilot whale trailing edge	Long‐finned pilot whale	Common bottlenose dolphin	Common dolphin	Dwarf sperm whale	Pygmy sperm whale (24)	Striped dolphin (7)	Risso's dolphin (7)
−2	NA	NA	NA	217	NA	335	NA	NA	NA	NA	NA	NA
2	NA	NA	NA	248	NA	338	NA	NA	NA	NA	NA	NA
4	NA	196	NA	49	NA	84	329	92	314	NA	246	NA
6	NA	82	222	*23 (24)*	340	39	159	45	160	246	110	233
8	283	44	86	14	169	24	90	*24 (66)*	86	154	65	123
10	182	30	49	9	81	14	*61 (331)*	17	58	95	45	77
12	*100 (86)*	22	29	6	54	*10 (8)*	45	13	39	64	30	55
14	70	*16 (86)*	21	5	35	8	34	8	29	48	21	42
16	51	12	12	4	25	6	25	6	23	37	17	34
18	38	10	8	3	19	5	21	5	19	30	15	25
20	31	8	6	2	13	4	16	4	17	23	12	22
22	23	7	4	2	9	3	16	4	14	20	9	19
24	21	6	3	2	7	3	13	3	11	18	8	17
26	16	5	*2 (24)*	2	5	2	10	3	9	13	7	13
28	1	4	2	2	4	2	9	2	*8 (6)*	14	6	14
40	4	2	2	2	*2 (8)*	2	4	2	4	7	3	6

*Note*: Numbers in italics reflect the sample size needed to detect a shift 95% of the time for the approximate value of the observed slope for each species that showed a significant poleward shift (Table 2), while accompanying numbers in brackets reflect the actual annual sample size for that species. No poleward shift was detected for pygmy sperm whales, striped dolphins and Risso's dolphins (annual sample sizes shown in column headers).

**FIGURE 5:**
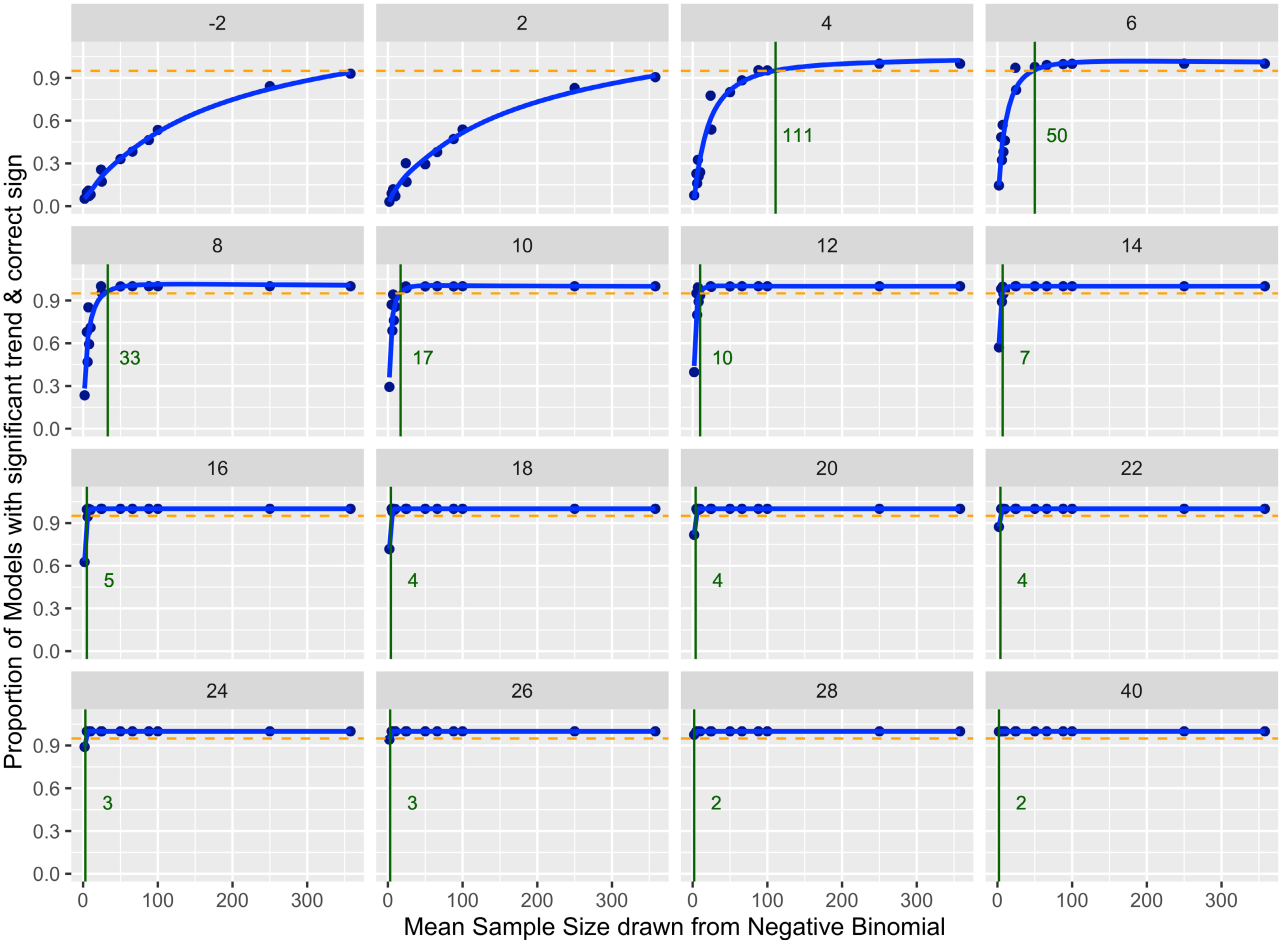
Proportion of 1000 model runs with a significant trend (*p*‐value <.05) and a slope estimate with the correct sign compared to the corresponding true slope for simulated trends in changes in the poleward distance through time, shown here for common dolphins. True slope values are indicated in gray at the top of each panel. Simulated data shown are based on observed variance in common dolphin stranding location and annual stranding event sample sizes (*n* = 2, 5, 6, 7, 8, 10, 24, 25, 50, 66, 86, 100, 250, 331), with slope values ranging from −2 to 40 km year^−1^. The green line and number indicates the minimum sample size necessary to identify a significant slope estimate of the correct sign 95% of the time, whereas the orange dotted line indicates a value of 0.95.

Results of the simulation analyses showed that mean annual sample size (number of stranding events per year) necessary to detect a significant poleward shift for a given slope varied considerably between species. Smaller sample sizes were needed to detect poleward shifts with higher slopes (Figure 5; Table 3). For example, for poleward shifts ≥16 km year^−1^, 4 and 6 strandings per year, respectively, were required to detect a significant poleward shift in the center of distribution for Atlantic white‐sided dolphins and common dolphins. Species with low mean annual variance in poleward distance such as Atlantic white‐sided dolphins, long‐finned pilot whales and common dolphins (Table 1) required a comparatively small sample size to detect a shift in the center of distribution with a given slope than species with a high variance such as Risso's dolphins or pygmy sperm whales. For example, based on slopes of the observed poleward shifts, a significant poleward shift in the center of distribution could be detected 95% of the time with as few as 17–24 strandings per year for common dolphins (observed slope of 8.7 km year^−1^). In contrast, approximately 77–154 strandings or more would be required to detect poleward shifts at this slope for Risso's dolphins and pygmy sperm whales. The results of the simulations suggested that for striped dolphins, Risso's dolphins, and pygmy sperm whales, there were fewer stranding events observed than the number required to detect a significant shift 95% of the time unless slopes were greater than approximately 20, 26 and 40 km year^−1^, respectively (Tables 1 and 3). No significant poleward shift was observed for these species. Species with low observed numbers of annual strandings such as long‐finned pilot whales and dwarf sperm whales generally showed wider CIs from the bootstrap analysis (Table 1). Species with a larger sample size, particularly those with a relatively low variance in poleward distance such as harbor porpoises, had more narrow CIs around the observed slope estimates.

Census data indicated that most counties along the eastern seaboard of the United States demonstrated population increases from 2000 to 2020 (77.7%). The counties with the largest population increases generally occurred in the SEUS (Figure S6). Distance from Florida had a small negative linear effect on population change for the east coast of the United States as a whole, and within the range of poleward distances over which a distributional shift was observed for strandings of long‐finned pilot whales, bottlenose dolphins and dwarf sperm whales (Figure S7; Table S2). The census data showed the opposite pattern that we would expect if changes to human population size were responsible for observed poleward shifts in odontocete stranding data. Strandings of multiple odontocete species occurred further north through time, with shifts for different species occurring across different poleward distances (Figure 3), while human population either showed no significant spatial trend, or human population changes were negatively associated with poleward distance.

## 4 DISCUSSION

The stranding dataset along the long coastline of the eastern seaboard of the United States, covering approximately 20° of latitude, provides a compelling scenario for examining changes to odontocete distributions in relation to climate‐driven oceanographic change. Waters of the NEUS have warmed dramatically in recent years, allowing stranding data to be assessed relative to rapid, directional climate change. Our results demonstrate marked shifts in the spatial distribution of strandings of cool water odontocete species linked with rapidly warming waters in the NEUS, and suggest that the distribution of these species is shifting north, out of the NEUS. Observed poleward shifts from 1996 to 2020 indicate that the center of distribution for strandings of cool water odontocete species was at or approaching the northern extent of the NEUS at the end of this study period. Furthermore, our observation that the center and trailing edge of the distribution of strandings for long‐finned pilot whales and Atlantic white‐sided dolphins converged in recent years suggests that the center of distribution may underestimate recent shifts in distribution out of US waters. The center of distribution was calculated using only data from the NEUS given the availability of consistently recorded data, and likely did not reflect the true center of distribution as the species shifted north out of the NEUS. The proportion of cool water odontocete species declined through time and was strongly linked with SST anomaly in the NEUS, providing further support for poleward shifts out of this region in association with rapid warming. Species‐level assessments suggested that declines in strandings of cool water species were driven by declines in the relative abundance of harbor porpoise, long‐finned pilot whale, and to a lesser extent Atlantic white‐sided dolphin strandings. Increases in warm water species were driven by common dolphin strandings in the NEUS, as well as bottlenose dolphins. The relative abundance of strandings for a particular species could be influenced by changes to the population size of those species, but due to insufficient data and changes in methodology, trends in abundance for our focal species cannot be determined (Hayes et al., 2021).

We assessed changes to odontocete species composition and poleward shifts in the distribution of odontocete stranding events and their associations with climate‐driven warming in the NEUS. Importantly, stranding events only allow us to make inferences about changes to odontocete distribution along the coastline and cannot account for changes such as shifts into offshore waters. A shift into offshore waters could make strandings less likely to be observed on land (Prado et al., 2013), and thus a shift into offshore habitats in the southern portion of a species range, but not further north, could lead to a decrease in stranding events observed in the south. Similarly, anthropogenic factors such as entanglement in fishing nets or the ingestion of plastics or other marine debris can lead to cetacean death and subsequent stranding (Kirkwood et al., 1997; Simmonds, 2012) and the spatial distribution of these factors could influence observed spatial patterns of strandings. However, poleward shifts in stranding events were observed at a range of rates, across a wide range of poleward distances (Figure 3), and across species with varying spatial distributions. It is therefore difficult to conceive of a mechanism through which offshore shifts in habitat use or anthropogenic factors could explain the observed patterns of poleward shifts. Shifts into offshore waters would also be unlikely to explain observed poleward shifts for harbor porpoises, which primarily occur in coastal areas (Embling et al., 2010; Hamazaki, 2002; Read & Westgate, 1997). Furthermore, in the NEUS, our observations that strandings of cool water species declined and contracted at the southern end of their range while strandings of warm water species increased and moved north suggests that these trends are driven by climate‐driven oceanographic change. Such opposing responses in warm‐ and cool‐water species within a community are considered diagnostic fingerprints that provide key evidence of the importance of climate change as a driver of biological pattern (Poloczanska et al., 2013). Together, the poleward shifts and changes to the relative abundance of odontocete species in the NEUS suggest a reorganization of the odontocete community in response to rapid climate‐driven warming.

We observed poleward shifts in strandings of all odontocete species for which simulations indicated there were sufficient data to model distribution changes. Using trailing edge values for cool water species, our estimates of poleward shifts for odontocete species along the eastern seaboard of the United States ranged from approximately 9 to 40 km year^−1^ (Table 1). These values exceeded all values of climate velocity observed in the SEUS (Figure 1c; mean −0.65 km year^−1^, 75% quantile 0.22 km year^−1^) and exceeded climate velocity typically observed in the NEUS (mean and 75% quantile of 6.15 and 7.5 km year^−1^, respectively), indicating that shifts were occurring faster than would be expected based on temperature. Recent studies suggest that most range shifts in marine species track changes in temperature (Lenoir et al., 2020; Pinsky et al., 2013; Poloczanska et al., 2013), but some ranges do not shift poleward as predicted in response to the rapid warming in the NEUS, highlighting that temperature alone cannot explain distributional shifts for marine species (Fredston et al., 2021). There is considerable variability in species' range shifts in response to climate change, even within particular regions (Donelson et al., 2019; Sunday et al., 2015), but our finding that observed shifts in odontocete species were consistently higher than predicted by climate velocity is striking. Ocean warming is typically considered to impact marine mammals indirectly through impacts on productivity and prey availability as warming is expected to have greater impacts on ectothermic species such as fish and invertebrates (Albouy et al., 2020; Gibson‐Reinemer et al., 2015; Sheldon et al., 2011; Sydeman et al., 2015). However, the distribution of marine mammals is constrained by thermal tolerances at broad spatial scales (Yeates & Houser, 2008), and marine mammal species with narrow thermal ranges are more often reported to be affected by climate change (Orgeret et al., 2022). Observations from strandings and fisheries bycatch indicate that the distribution of long‐finned pilot whales shifted poleward more rapidly than their prey species in response to warming waters in the NEUS, suggesting a direct response to warming rather than an indirect effect mediated by climate impacts on prey species (Thorne & Nye, 2021). Species traits likely play an important role in climate shifts (Chen et al., 2011; Donelson et al., 2019; MacLean & Beissinger, 2017; Sunday et al., 2015), and generalist species with better swimming abilities and a larger latitudinal range may show greater range shifts in response to climate warming (Sunday et al., 2015). As highly mobile species with expansive ranges, odontocetes may thus be expected to show greater distributional shifts than their prey species (Thorne & Nye, 2021). Furthermore, the focal odontocete species in our analyses are generalist consumers (Smith et al., 2015; Staudinger et al., 2014) and can target different prey species when prey abundance varies in space or time (Santos et al., 2004, 2014). Together these findings suggest large‐scale and rapid shifts in odontocete distribution occurring across international boundaries that may disrupt species interactions in the NEUS. Such shifts will have important implications for management, as these changes could influence the spatial location of risk from anthropogenic threats. For example, long‐finned pilot whales are caught as bycatch in the bottom trawl fishery in the NEUS (Chavez‐Rosales et al., 2018). Our results suggest that poleward shifts in long‐finned pilot whales could lead to less frequent fisheries interactions in US waters but that interactions might be expected to become more frequent in Canadian waters as long‐finned pilot whale habitat shifts north out of US waters.

The observed increase in common dolphin strandings in the NEUS in association with warming waters suggests an important change in the species composition of odontocetes in this region (Figure S3; Table S2). Whereas odontocete strandings in the NEUS were dominated by harbor porpoises in the late 1990s with very few common dolphin strandings, strandings in the NEUS have been dominated by common dolphins in recent years, particularly in the Gulf of Maine, followed by Southern New England (Figure S4). While we observed a poleward shift in the distribution of strandings of common dolphins, this shift was not significant following adjustment for multiple comparisons of shifts in the distribution of strandings for different odontocete species. In this case, the observed increase in the proportion of common dolphin strandings in the NEUS cannot be explained by the observed poleward shift in the distribution of strandings as the vast majority of common dolphin strandings occurred in the NEUS throughout the study period. SST has increased most rapidly in the Gulf of Maine, followed by Southern New England (Figure S2), mirroring increases in common dolphin strandings. Common dolphins are associated with warm waters and Gulf Stream features that typically occur on the outer continental shelf of the NEUS (Hayes et al., 2021; Selzer & Payne, 1988; Waring et al., 1992), and changes to Gulf Stream dynamics in recent years may have influenced observed patterns of common dolphin strandings. A shift in the position of the Gulf Stream has been linked with abrupt warming observed on the continental shelf in the Gulf of Maine since 2009–2010 and an associated ecosystem shift (Gonçalves Neto et al., 2021). In addition, warm core rings, which bring warm, nutrient‐rich water onto the shelf, have formed more frequently since 2000 (Gangopadhyay et al., 2019, 2020). The ring footprint index, reflecting the area of warm core rings on the continental slope, also increased significantly after 2000, with the highest increase observed between 70 and 65° W (Gangopadhyay et al., 2020), which covers the northern reaches of the NEUS. Thus, common dolphins may have occurred more frequently in more northerly regions of the NEUS in recent years due to Gulf Stream changes and associated patterns of warming. Historical data indicate that common dolphins occurred further south along the eastern seaboard of the United States in the 1960s than they do today (Jefferson et al., 2009) and long‐term warming in the NEUS may be linked with continued poleward shifts in this species.

The contrasting oceanographic trends in the NEUS, which showed rapid warming, and the SEUS, which showed no warming through time, offer a unique opportunity to assess the impacts of regional climate change on taxa with different thermal preferences. MacLeod (2009) postulated that the range of warm water species would expand in response to warming waters, while the range of cool water species would shift poleward. Poleward shifts were observed in both cool water odontocetes and warm water odontocetes that primarily occurred in the SEUS but whose ranges extended into the NEUS, suggesting that warming in the NEUS caused these species to shift further north into waters of the NEUS. Although we found some evidence for an increase in the relative abundance of warm water species and a decrease in relative abundance of cool water species in the SEUS through time, these patterns were not linked with SST anomaly. SST showed no warming trend in the SEUS, and thus changes in the relative abundance of cool and warm water species in this region may be attributable to the rapid warming observed in the NEUS. Our findings indicate that warming waters in the NEUS had particularly marked impacts on odontocete species with trailing edge distributions in this region, indicated by both the rapid poleward shifts observed and declines in the relative abundance of trailing edge species. Poloczanska et al. (2013) observed faster shifts in the leading edge of distributions of marine organisms but suggested that these more rapid shifts were driven by stronger regional warming. Similarly, the stronger responses observed in the present study for cool water species for which trailing edge distributions occurred in the NEUS may be due to the rapid warming in this region.

Historically, Cape Hatteras served as an important ecogeographic barrier along the eastern seaboard of the United States, separating cool and warm water species (Calder, 1992; Hutchins, 1947; Pappalardo et al., 2015; Roy et al., 2012). While the trailing edge of the distribution of harbor porpoises, Atlantic white‐sided dolphins and long‐finned pilot whales historically occurred at or near Cape Hatteras, our results suggest that the trailing edge of their distributions has shifted north as waters in the NEUS have warmed (Figures 3 and 4). Future studies could assess whether these changes are part of a broader pattern in which the range of cool water marine species contracts poleward and alters the location or importance of this ecogeographic barrier.

Assessing distributional shifts is key to understanding the biological impacts of climate‐driven warming, but sample sizes may limit the ability to detect and quantify shifts. In the present study, simulation analyses provided a useful means of assessing the statistical power of observed poleward shifts in the distribution of odontocete strandings. Our results highlighted that for some odontocete species, poleward distributional shifts can be assessed using a relatively small number of strandings depending on the speed of the shift and the spatial variability of the species' distribution. Simulations suggested that given the observed sample sizes for three of the focal warm water species (pygmy sperm whales, striped dolphins and Risso's dolphins), poleward shifts would not have been detectable unless rapid shifts of approximately 22 km year^−1^ (pygmy sperm whales and striped dolphins), or 40 km year^−1^ (Risso's dolphins) occurred. Thus, it is not possible to determine whether these species are not shifting in response to warming waters or whether they are shifting at more moderate rates that could not be detected with the available data.

Our findings highlight the utility of stranding records for assessing broad changes in the distribution of cetacean species and species composition of cetacean communities. Cetacean distribution is typically assessed using data from at‐sea surveys, which are both expensive and labor intensive. Because cetacean species occur at low densities and have wide distributions, effort for standardized at‐sea surveys is often too low and does not cover sufficient spatiotemporal scales to allow changes to the abundance or distribution of cetacean populations to be detected (Jewell et al., 2012; Kaschner et al., 2012; Taylor et al., 2007). In contrast, stranding records can provide data over large spatial scales, are considerably less expensive to collect, and are largely independent of spatial and temporal sampling (Pyenson, 2011). Records from stranding events can be particularly useful for examining species‐specific trends in species such as short‐ and long‐finned pilot whales which differ considerably in their ecology but are often analyzed together since they are difficult to distinguish at sea (Thorne & Nye, 2021). Several countries that have had standardized stranding programs in place for many years have long time series that can be used to assess changes to cetacean communities through time. For example, Australia and New Zealand have long coastlines, stranding records that stretch over multiple decades (Pyenson, 2011), and cover a wide range of latitudes. Both South East and South West Australia were identified as global marine hotspots based on 50 years of SST data (Hobday & Pecl, 2014) suggesting that stranding records from these regions could provide important knowledge of climate impacts on cetaceans (Evans et al., 2005). However, using strandings to observe changes in cetacean distribution may not be feasible in many sparsely populated high‐latitude regions of interest in the Northern hemisphere, such as Greenland and the Canadian Arctic.

We assessed whether changes to the human population along the eastern seaboard of the United States might have contributed to observed patterns of strandings, and we did not find evidence suggesting that this was the case. However, studies assessing trends in the distribution of strandings should consider patterns of human use of coastal regions, particularly for more fine‐scale analyses. The widespread use of technological devices such as smartphones and georeferenced cameras has produced huge quantities of data that can be used to assess patterns of human movement and behavior (de Arriba‐Pérez et al., 2016; Simini et al., 2012, 2013; Sinclair et al., 2018; Thums et al., 2018). Future studies could use these crowd‐sourced data to assess or account for changes to human use of coastal regions when studying patterns of cetacean strandings.

Our approach focuses on broad, long‐term trends in odontocete distribution and species composition to understand the impacts of warming waters in the NEUS on cetaceans. Lower frequency environmental effects are also important to understanding environmental drivers of cetacean distribution, and stranding data may also be useful in assessing smaller‐scale patterns. For example, the proportion of cool water odontocete species occurring in the NEUS suggested a periodic increase in these species occurring every 3–6 years (Figure 2a) and may be caused by oceanographic variability. Furthermore, while our analysis focuses specifically on changes to SST given the rapid warming occurring in the NEUS, other dynamic environmental variables are important determinants of cetacean distribution and may be key to understanding distributional changes in cetaceans in other regions. For example, Warlick et al. (2022) found links between the Pacific Decadal Oscillation and strandings of cetacean species in the US Pacific Northwest, while Evans et al. (2005) found that large‐scale climatic events increasing biological productivity influences patterns of cetacean strandings in southeast Australia.

## 5 CONCLUSIONS

Together, our findings suggest widespread impacts of rapid warming on the distribution and species composition of odontocetes in the NEUS. Changes in the relative abundance of strandings suggest that warm water species have become more abundant in the NEUS as cool water species have shifted north out of the NEUS and have become less abundant. Changes to the cetacean community were less apparent in the SEUS, highlighting the importance of regional warming in driving changes to the cetacean community along the eastern seaboard of the United States. Simulations provided helpful context for understanding data required to demonstrate poleward shifts in distributions under climate change. Our results demonstrate the utility of the stranding record for understanding climate impacts on the relative abundance and community structure of cetaceans given limitations of at‐sea survey effort and modeling approaches for predicting distributions under rapidly changing conditions.

## CONFLICT OF INTEREST

The authors declare no conflict of interest.

## Supporting information


Appendix S1

## Data Availability

The data that support the findings of this study are openly available via the National Stranding Database Public Access at https://www.fisheries.noaa.gov/national/marine‐life‐distress/national‐stranding‐database‐public‐access#:~:text=Includes%20information%20on%20every%20stranded%2Fdetermination%2C%20and%20other%20information. Sea surface temperature data is available at https://psl.noaa.gov/data/gridded/data.noaa.oisst.v2.highres.html.
