# Supplementary material for: Rapid restructuring of the odontocete community in an ocean warming hotspot
Source: Glob Chang Biol. 2022 Aug 23;28(22):6524–40. doi: 10.1111/gcb.16382 (PMC9804436; doi:10.1111/gcb.16382)
Supplement: Supplementary file 1 — Appendix S1 [file GCB-28-6524-s001.docx]

**Supplementary Information for “****Rapid restructuring of the odontocete community in an ocean warming hotspot”**

*Processing, standardizing and assessing locations of stranding records*

We assessed the accuracy of stranding location records by cross-checking latitude and longitude locations with locations geocoded using descriptive location metadata (e.g. City, County, State and location descriptors). We used the *tidygeocoder* package in R (Cambon et al. 2021) and queried Open Street Map dataset to geocode each stranding record (i.e., determine the location of known geographies) using city, county, and state data described above. The resulting dataset included 19,260 stranding records of individual animals, each with two sets of coordinates: the recorded latitude and longitude coordinates and the geocoded coordinates generated from the descriptive location metadata. A small number of records did not have sufficient information for geocoding and cross-checking latitude and longitude coordinates and were excluded (n = 3 records). We calculated the distance between the recorded and the geocoded coordinates to cross-check the recorded locations and used the recorded latitude and longitude coordinates for all records for which this distance was < 50 km (n = 18,366) given the spatial scale of this analysis (poleward distances ranged from 0 to approximately 3500 km). The recorded locations for the remaining records were verified manually through spatial plots (n = 358). Where recorded coordinates were nonsensical (e.g., far inland from any shoreline), the descriptive city, county and state data were assumed to be correct and the corresponding geolocated coordinates were used in analyses (n = 536).

Stranding events can consist of a single individual or dozens of individuals, and stranding events involving more than 2 individuals are labelled as mass strandings in the MMHSRP. There is a great deal of variability between species in the tendency to mass strand, and species that mass strand also frequently strand individually. Mass strandings could inflate abundance or occurrence estimates at a particular location if each individual was considered separately since the whole social group, rather than one individual, may strand if one individual is affected (Geraci 1978, Rogan et al. 1997, Mazzariol et al. 2018). Previous studies have found that stranding events, which can consist of one or multiple individuals, reflect abundance and richness in cetacean communities (Maldini et al. 2005, Pyenson 2010). We therefore assessed changes in the species composition and location of stranding events. Strandings of the same species occurring on the same day (identified as a mass stranding or cow/ calf pair in the MMHSRP) were considered to be part of the same stranding event when assessing temporal trends in strandings. We also accounted for Unusual Mortality Events (UMEs), defined by the Marine Mammal Protection Act (16 U.S.C. 1361) as strandings that are unexpected, involve a significant die-off of any marine mammal population; and demand immediate response. UMEs are identified by the Working Group on Marine Mammal Unusual Mortality Events, established by NFMS (<https://www.fisheries.noaa.gov/national/marine-mammal-protection/marine-mammal-unusual-mortality-events>). The occurrence of UMEs could inflate the number of stranding events for a particular species in a given year, and could thus influence our analysis on the relative occurrence of strandings of different species or climatic groups in the NEUS and SEUS, and we therefore removed any strandings identified as UMEs in the stranding database from analyses.

*Simulating effects of sample size, variance, and slope on the detection of poleward distributional shifts*

Based on observed numbers of stranding events (Supplemental Table 1) and slopes observed in each species’ linear model assessing poleward shifts, we assessed sample sizes of n=2, 5, 6, 7, 8,10, 24, 25, 50, 66, 88, 100, 250, and 358 to represent mean stranding events observed each year, and slope values 𝛽 = -2 to 28 km/ year at intervals of 2, as well as 40 km/ year to reflect the maximum slope observed. Simulations were conducted separately for each species using the variance in poleward distance calculated from observed stranding locations of that species. A subset of these scenarios represent observed mean annual stranding event sample size values for our candidate species (Table 1). For each scenario, we generated 1000 simulations, with each of the simulation representing annual stranding event sample sizes for 25 years drawn from a negative binomial distribution centered on the mean sample size scenarios described above and a dispersion parameter of 3. We used a standard linear regression likelihood function to obtain μ_nbt_ the mean annual expected poleward distance for each slope (β_b_), sample size scenario (n), and year (t) (Equation 1).

Equation 1 $\mu_{nbt}\sim\beta_{0} + \beta_{nb}({year}_{t})+ {}_{nbt}$

Using μ_nbt ,_we then randomly drew along-shelf distance values from either a normal distribution or Weibull distribution depending on the fitted distribution of observed distances for each species and the observed standard deviation. Standard deviation values were drawn from a gamma distribution with shape equal to the mean annual standard deviation in poleward distance observed for each species and scale = 1 for each simulation. For each dataset we fit a linear model to the simulated along-shelf distances as a function of time. For each species, slope, and sample size scenario, we assessed the proportion of the 1000 model runs which returned a significant trend (p-value < 0.05) and a slope estimate with the correct sign compared to the corresponding true slope from which the underlying data were generated. In addition, we assessed how 95^th^ percentile of the modeled slope estimates from the 1000 simulations varied with sample size.

**Supplementary Figure 1:** Range maps of odontocete species that were assessed using power analysis simulations. Data from IUCN (2021). The study area is shown in red in the first panel. Note that limits to spatial ranges of odontocetes are not well understood, and that our analysis and discussion suggest some differences from the general ranges shown here (e.g., our analysis suggests that long-finned pilot whales no longer occur as far south as indicated here, while Atlantic white-sided dolphins were historically observed further south than indicated here).


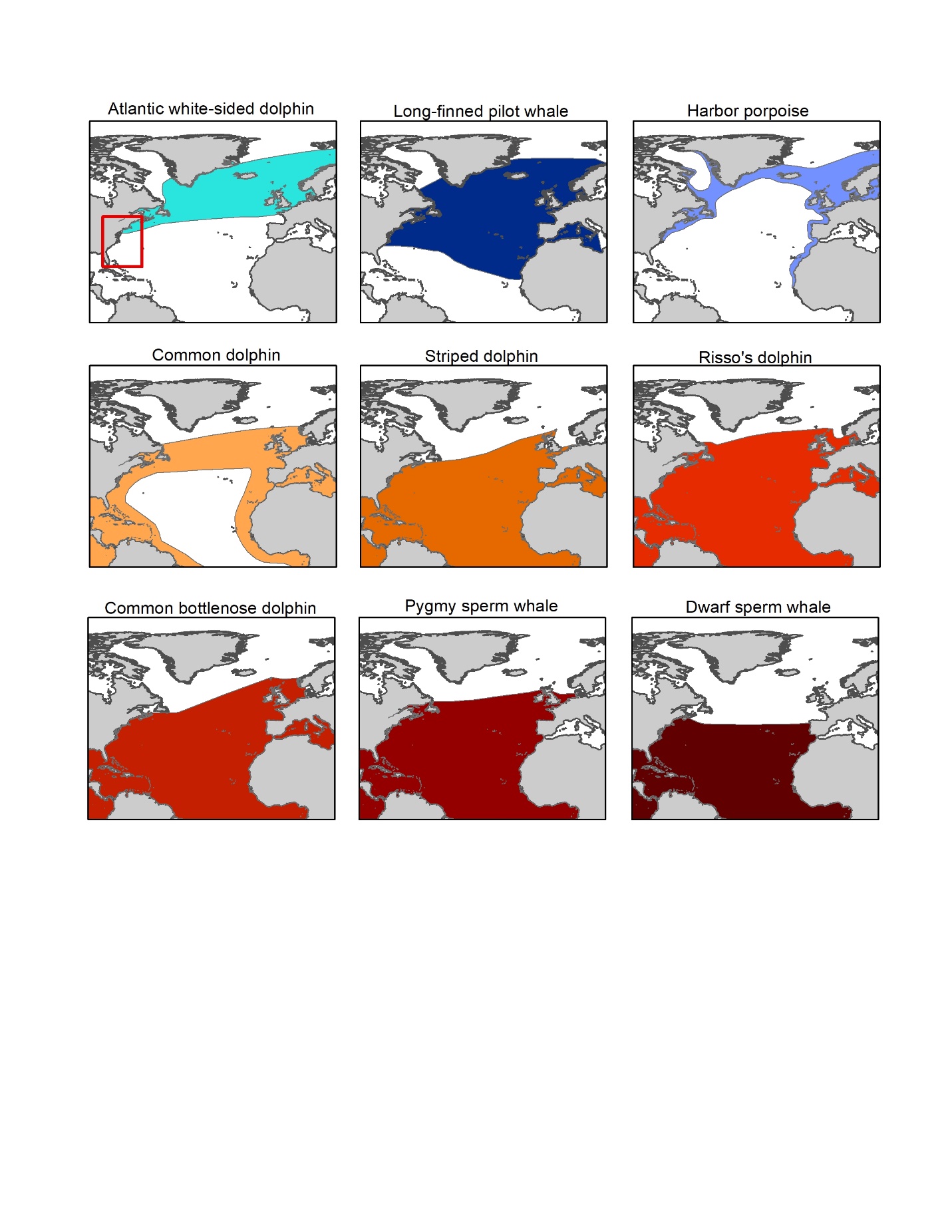


**Supplementary Figure 2**: Temporal gradient (℃ yr-1) in sea surface temperature (SST) in the Gulf of Maine, Southern New England and Mid-Atlantic Bight, respectively, from 1996-2020.


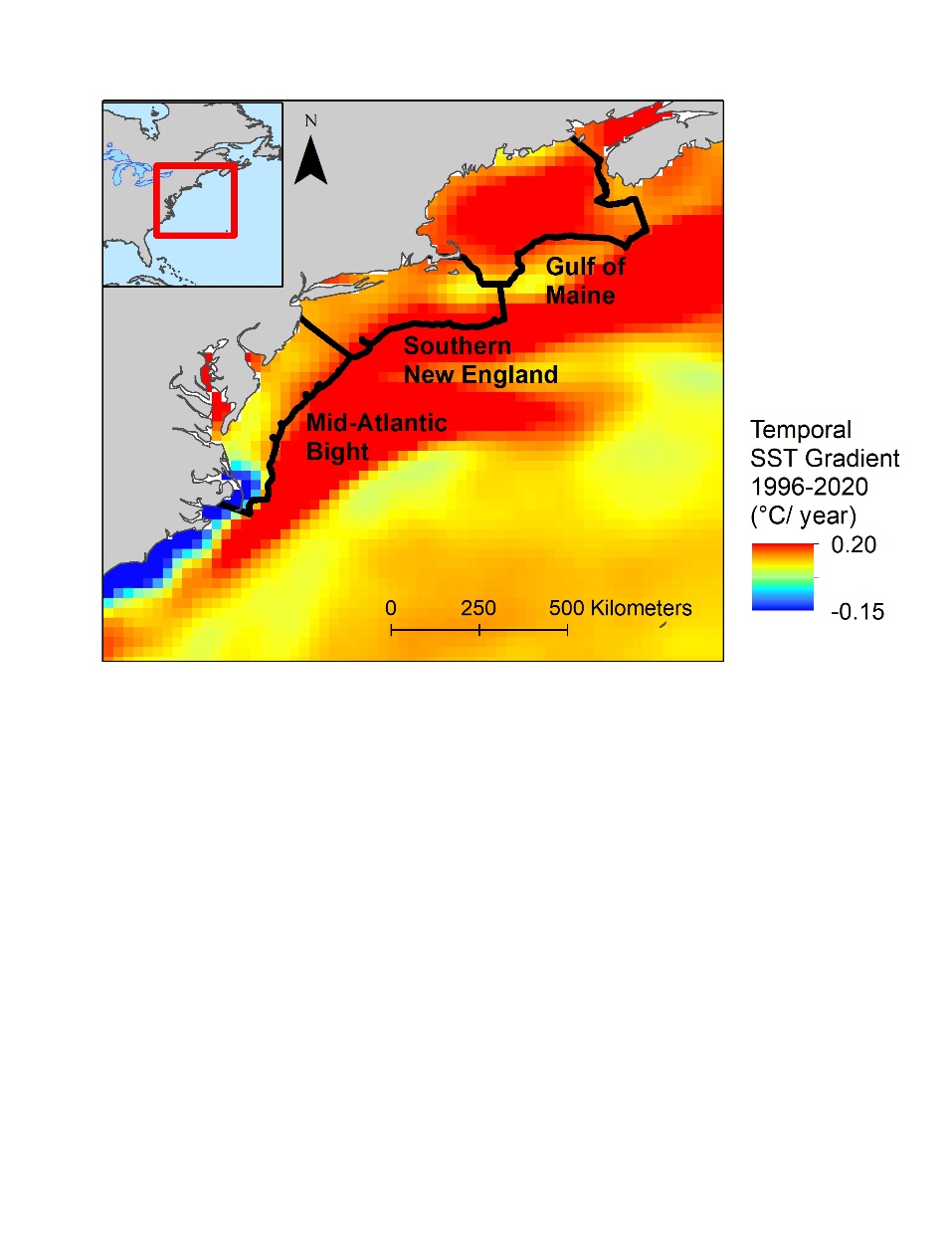


**Supplementary Figure 3:** Changes in the proportion of odontocete strandings in the Northeast United States by species on an annual basis relative to sea surface temperature anomaly from 1996-2020. Only species comprising at least 5% of strandings at the beginning of the study period are shown.


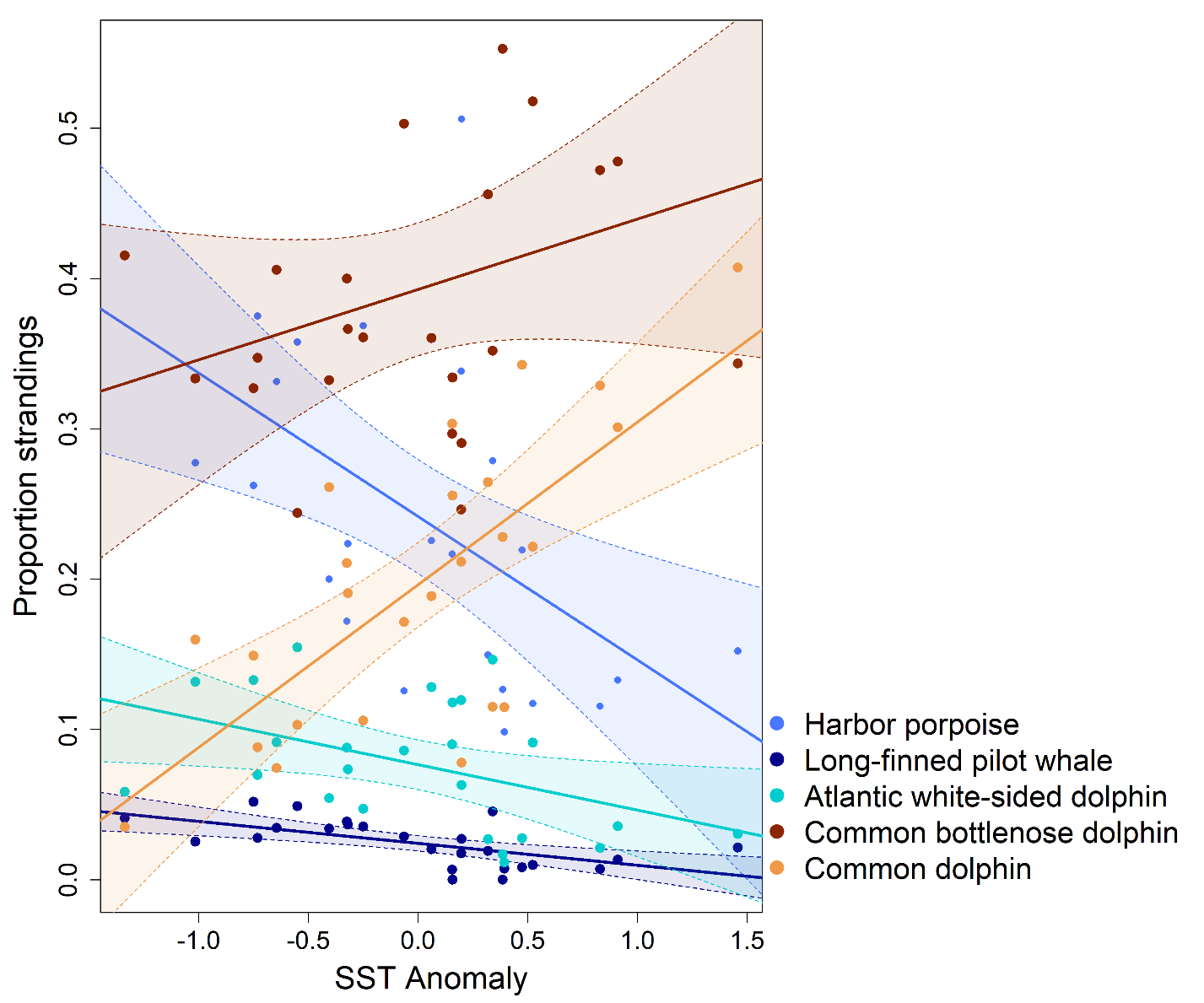


**Supplementary Figure 4**: Changes in the proportion of odontocete strandings in the Gulf of Maine, Southern New England and Mid-Atlantic Bight, respectively, by species on an annual basis from 1996-2020. Only species comprising at least 5% of strandings at the beginning of the study period are shown.


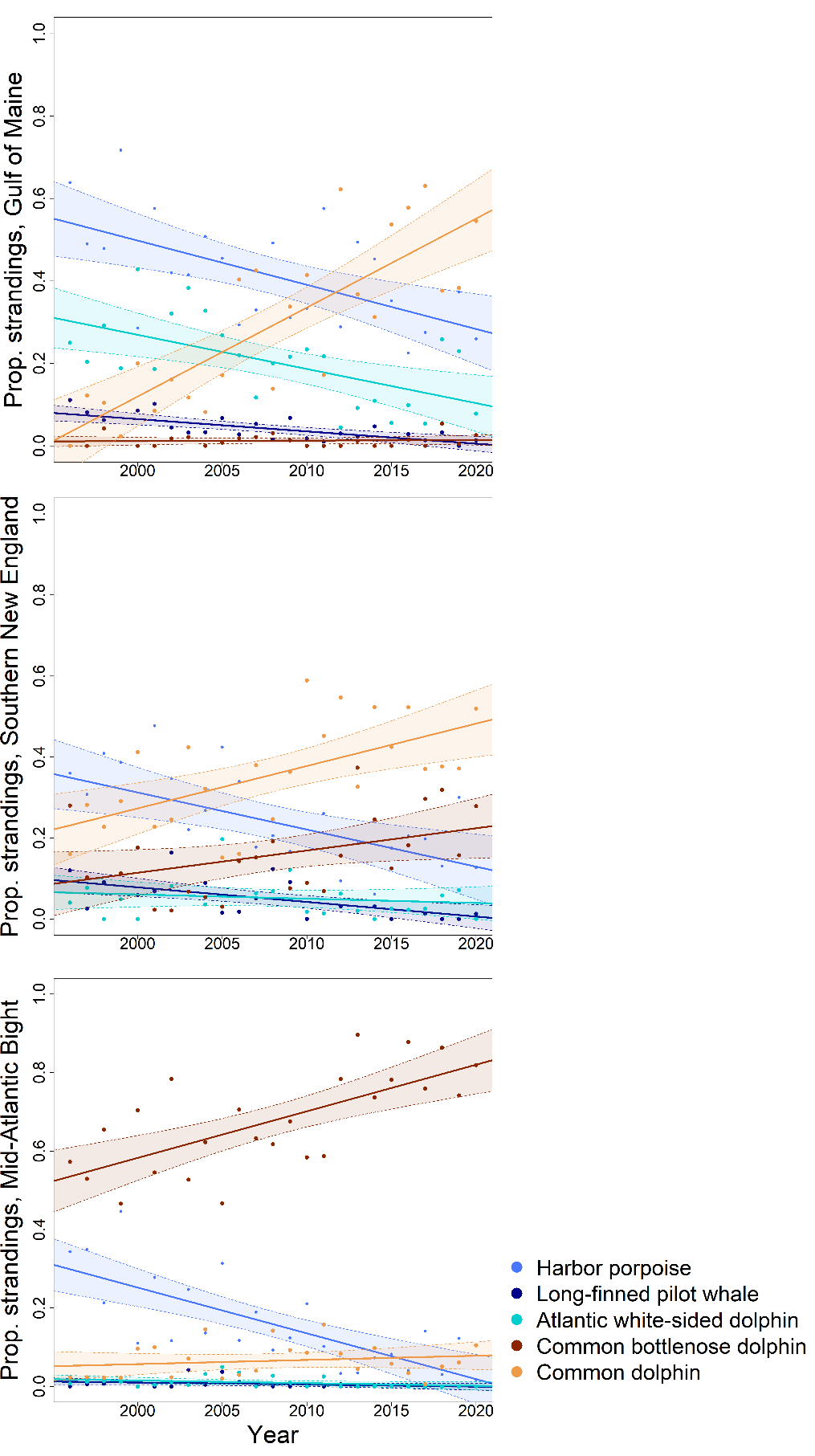


**
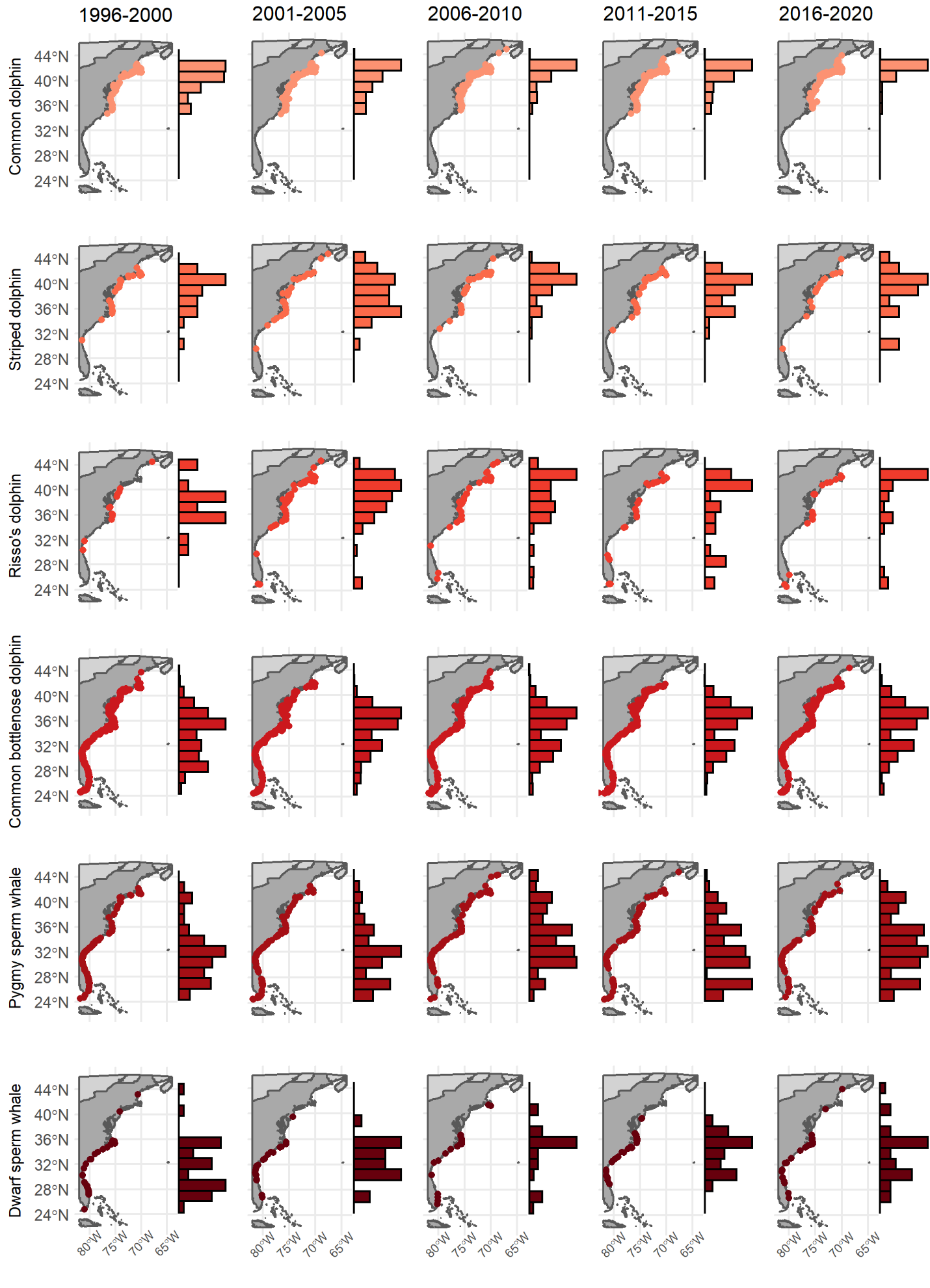
Supplementary Figure 5:** Changes in the spatial distribution of the stranding events of six warm water odontocete species along the eastern seaboard of the US that were assessed using power analysis simulations, shown in five-year periods. Histograms reflect the proportion of stranding events for each species and time period binned based on latitude.

**Supplementary Figure 6**: Change in the human population of counties along the eastern seaboard of the United States from 2000-2020. Data from census.gov


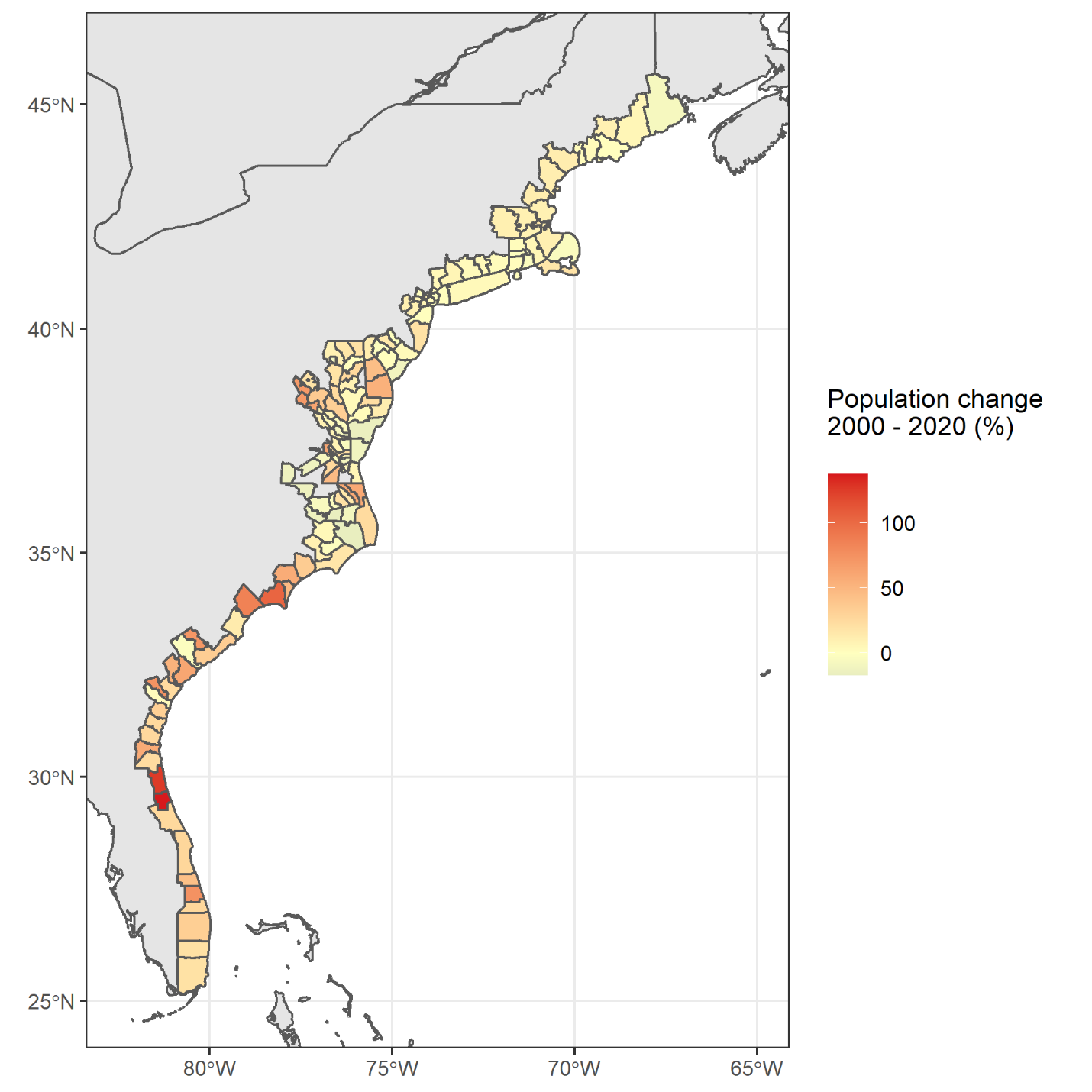


**Supplementary Figure 7**: Change in the human population of counties along the eastern seaboard of the United States from 2000-2020 relative to poleward distance (along-shelf distance from the southern tip of Florida). Population changes are shown for poleward distances across which odontocete species showed poleward shifts. Distance from Florida had a small negative linear effect on population change within the range of poleward distances over which a distributional shift was observed for strandings of long-finned pilot whales, bottlenose dolphins and dwarf sperm whales.

**
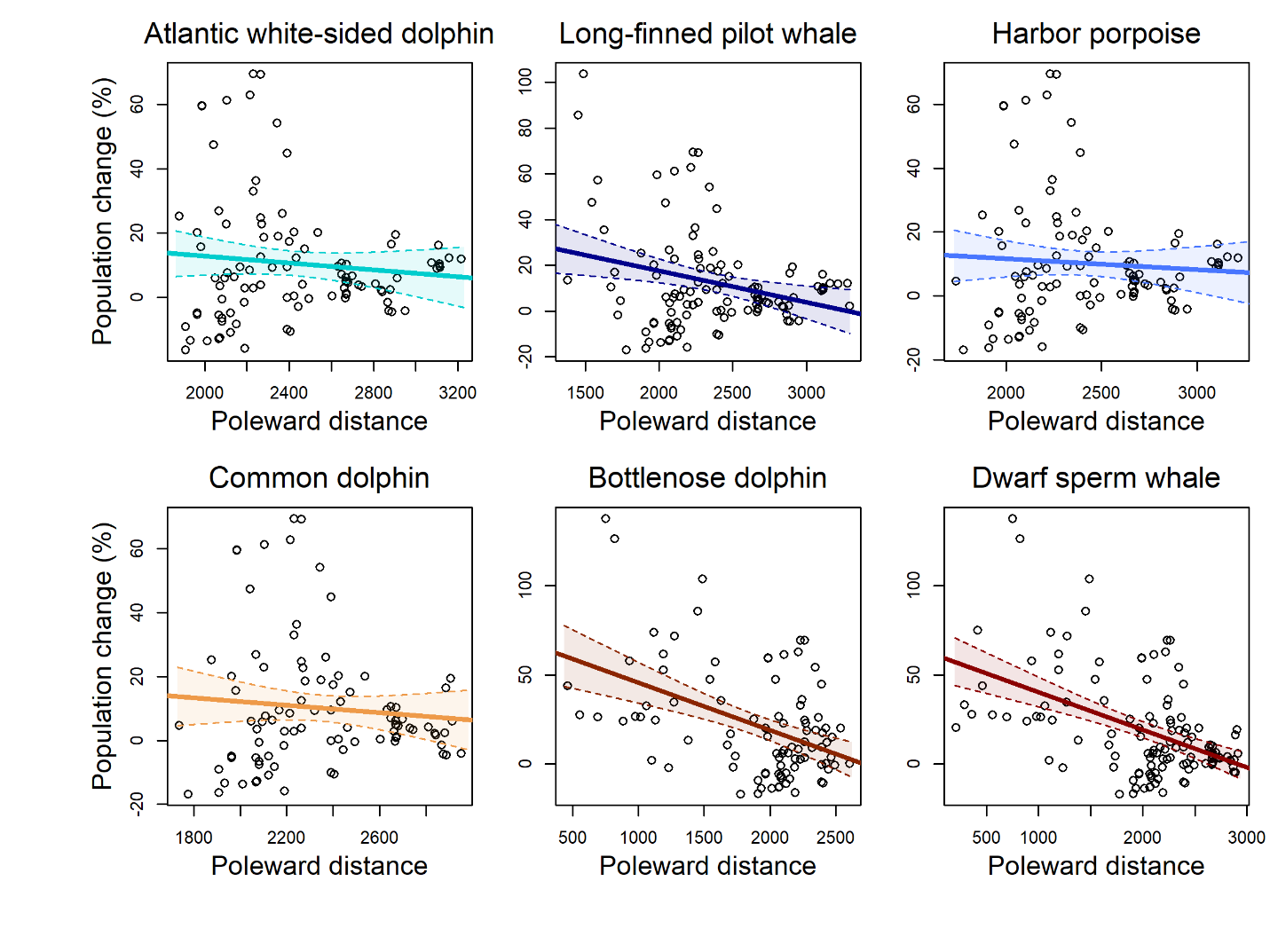
Supplementary Table 1:** Number of stranding events and climatic groupings for odontocete species identified to species level in the strandings record in the Northeast and Southeast US from 1996-2020. Species groupings are taken from MacLeod (2009), though we use the terms “cool water species” (rather than cool and warm water limited), “warm water species” (rather than warm water limited), and Arctic/ Sub-Arctic (rather than cool water limited) for simplicity given the North Atlantic focus of the study. Unidentified Kogia are also included here and in analyses of cetaceans by climatic grouping as both members of this genera, pygmy sperm whales (*Kogia breviceps*) and dwarf sperm whales (*K. sima*), are classified as warm water species.

| **Common name** | **Species name** | **Total stranding events** | **Mean stranding events per year** | **Climatic grouping** |
| --- | --- | --- | --- | --- |
| Whale, sperm | *Physeter catodon* | 75 | 3 | Cosmopolitan |
| Whale, beluga | *Delphinapterus leucas* | 3 | 0.12 | Arctic/ Sub-Arctic |
| Whale, northern bottlenose | *Hyperoodon ampullatus* | 3 | 0.12 | Arctic/ Sub-Arctic |
| Porpoise, harbor | *Phocoena phocoena* | 2157 | 86.28 | Cool Water |
| Dolphin, Atlantic white-sided | *Lagenorhynchus acutus* | 607 | 24.28 | Cool Water |
| Whale, pilot, long-finned | *Globicephala melas* | 182 | 7.28 | Cool Water |
| Dolphin, white-beaked | *Lagenorhynchus albirostris* | 19 | 0.76 | Cool Water |
| Whale, True's beaked | *Mesoplodon miras* | 17 | 0.68 | Cool Water |
| Whale, Sowerby's beaked | *Mesoplodon bidens* | 10 | 0.4 | Cool Water |
| Dolphin, Fraser's | *Lagenodelphis hosei* | 2 | 0.08 | Warm Water |
| Dolphin, common bottlenose | *Tursiops truncatus* | 8263 | 330.52 | Warm Water |
| Dolphin, common | *Delphinus delphis* | 1658 | 66.32 | Warm Water |
| Whale, pygmy sperm | *Kogia breviceps* | 599 | 23.96 | Warm Water |
| Dolphin, Risso's | *Grampus grisues* | 178 | 7.12 | Warm Water |
| Dolphin, striped | *Stenella coeruleoalba* | 170 | 6.8 | Warm Water |
| Whale, dwarf sperm | *Kogia sima* | 155 | 6.2 | Warm Water |
| Whale, pilot, short-finned | *Globicephala macrorhynchus* | 116 | 4.64 | Warm Water |
| Dolphin, Atlantic spotted | *Stenella frontalis* | 95 | 3.8 | Warm Water |
| Whale, Gervais' beaked | *Mesoplodon europaeus* | 81 | 3.24 | Warm Water |
| Whale, Cuvier's beaked | *Ziphius cavirostris* | 36 | 1.44 | Warm Water |
| Whale, Blainville's beaked | *Mesoplodon densirostris* | 23 | 0.92 | Warm Water |
| Whale, melon-headed | *Peponocephala electra* | 18 | 0.72 | Warm Water |
| Whale, pygmy killer | *Feresa attenuata* | 17 | 0.68 | Warm Water |
| Dolphin, pantropical spotted | *Stenella attenuata* | 16 | 0.64 | Warm Water |
| Dolphin, spinner | *Stenella longirostris* | 16 | 0.64 | Warm Water |
| Dolphin, rough-toothed | *Steno bredanensis* | 16 | 0.64 | Warm Water |
| Whale, false killer | *Pseudorca crassidens* | 10 | 0.4 | Warm Water |
| Whale, unidentified Kogia (dwarf/pygmy sperm whale) | *Kogia* spp. | 7 | 0.28 | Warm Water |
| Dolphin, clymene | *Stenella clymene* | 7 | 0.28 | Warm Water |

**Supplementary Table 2:** Estimated parameters of linear regressions used to evaluate changes in Sea Surface Temperature (SST) anomaly, the poleward distance of the center and trailing edge of distributions of odontocetes, and the proportion of warm water, cool water, cosmopolitan and Arctic/ sub-Arctic odontocete species from 1996 to 2020, as well as changes in human population from 2000 to 2020. Significant relationships are shown in bold, with the Dunn-Šidák adjusted α level of significance for multiple comparisons, α′, shown for each set of analyses. Relationships that were significant at α=0.05 are italicized. NEUS= Northeast United Statues, SEUS= Southeast United States.

| **Model** | **Estimate** | **Standard error** | **t value** | **Pr(> t)** | **Adjusted R^2^** |
| --- | --- | --- | --- | --- | --- |
| **SST Anomaly ~ year (α′ =1.27E-02)** |  |  |  |  |  |
| NEUS | **6.17E-02** | **1.28E-02** | **4.81** | **7.52E-05** | **0.48** |
| SEUS | -1.53E-02 | 1.10E-02 | -1.40 | 0.18 | 3.83E-02 |
|  |  |  |  |  |  |
| **Poleward distance ~ year (α′ = 5.68E-03)** | |  |  |  |  |
| Atlantic white-sided dolphin, trailing edge | **26.58** | **7.59** | **3.50** | **1.92E-03** | **0.32** |
| Atlantic white-sided dolphin | **5.23** | **1.58** | **3.32** | **3.13E-03** | **0.30** |
| Long-finned pilot whale, trailing edge | **40.55** | **11.31** | **3.59** | **1.74E-03** | **0.35** |
| Long-finned pilot whale | *13.05* | *4.93* | *2.65* | *1.51E-02* | *0.21* |
| Harbor porpoise, trailing edge | **11.19** | **3.83** | **2.92** | **4.71E-03** | **0.24** |
| Harbor porpoise | **14.03** | **3.25** | **4.32** | **2.54E-04** | **0.42** |
| Common dolphin | *8.72* | *3.06* | *2.85* | *9.09E-03* | *0.23* |
| Common bottlenose dolphin | **9.26** | **1.80** | **5.16** | **3.59E-05** | **0.53** |
| Dwarf sperm whale | **27.76** | **8.58** | **3.23** | **3.67E-03** | **0.28** |
|  |  |  |  |  |  |
| **Proportion of strandings in NEUS by category ~ year (α′ = 1.27E-03)** | | | |  |  |
| Warm water species | **1.29E-02** | **2.48E-03** | **5.19** | **2.93E-05** | **0.52** |
| Cool water species | **-1.28E-02** | **2.50E-03** | **-5.10** | **3.61E-05** | **0.51** |
| Arctic species | -4.40E-06 | 6.44E-05 | -0.07 | 0.95 | -4.33E-02 |
| Cosmopolitan species | -9.75E-05 | 9.86E-05 | -0.99 | 0.33 | -8.90E-04 |
|  |  |  |  |  |  |
| **Proportion of strandings in NEUS by category ~ SST anomaly (α′ = 1.27E-03)** | | | | |  |
| Warm water species | **1.30E-01** | **3.20E-02** | **4.05** | **4.98E-04** | **0.39** |
| Cool water species | **-1.30E-01** | **3.18E-02** | **-4.10** | **4.42E-04** | **0.40** |
| Arctic species | -1.89E-04 | 7.38E-04 | -0.26 | 8.00E-01 | -4.05E-02 |
| Cosmopolitan species | 1.03E-03 | 1.14E-03 | 0.91 | 0.37 | -7.49E-03 |
|  |  |  |  |  |  |
| **Proportion of strandings in SEUS by category ~ year (α′ = 1.70E-03)** | | | |  |  |
| Warm water species | *2.74E-03* | *1.10E-03* | *2.48* | *2.08E-02* | *0.18* |
| Cool water species | *-2.56E-03* | *1.12E-03* | *-2.27* | *3.26E-02* | *0.15* |
| Arctic species | NA | NA | NA | NA | NA |
| Cosmopolitan species | -1.75E-04 | 1.19E-04 | -1.48 | 0.15 | 4.67E-02 |
|  |  |  |  |  |  |
| **Proportion of strandings in SEUS by category ~ SST anomaly (α′ = 1.70E-03)** | | | | |  |
| Warm water species | -1.27E-04 | 2.27E-02 | -5.61E-03 | 1.00 | -4.35E-02 |
| Cool water species | 1.37E-03 | 2.27E-02 | 6.05E-02 | 0.95 | -4.33E-02 |
| Arctic species | NA | NA | NA | NA | NA |
| Cosmopolitan species | -7.96E-04 | 2.26E-03 | -0.35 | 0.73 | -3.79E-02 |
|  |  |  |  |  |  |
| **Proportion of all strandings in NEUS ~ year (α′ = 8.51E-03)** | | |  |  |  |
| Atlantic white-sided dolphin | -2.10E-03 | 1.16E-03 | -1.82 | 8.25E-02 | 8.73E-02 |
| Long-finned pilot whale | **-1.54E-03** | **2.86E-04** | **-5.39** | **1.77E-05** | **0.54** |
| Harbor porpoise | **-1.04E-02** | **2.19E-03** | **-4.73** | **9.19E-05** | **0.47** |
| Common dolphin | **1.03E-02** | **1.68E-03** | **6.10** | **3.23E-06** | **0.60** |
| Common bottlenose dolphin | 4.59E-03 | 2.93E-03 | 1.57 | 1.31E-01 | 5.70E-02 |
| Dwarf sperm whale | *1.18E-04* | *5.18E-05* | *2.27* | *3.28E-02* | *0.15* |
|  |  |  |  |  |  |
| **Proportion of all strandings in SEUS ~ year (α′ = 8.51E-03)** | | |  |  |  |
| Atlantic white-sided dolphin | -1.42E-04 | 1.45E-04 | -0.98 | 0.34 | -1.87E-03 |
| Long-finned pilot whale | *-1.60E-04* | *6.64E-05* | *-2.41* | *0.02* | *0.17* |
| Harbor porpoise | -2.27E-03 | 1.07E-03 | -2.11 | 0.05 | 0.13 |
| Common dolphin | 2.44E-04 | 3.62E-04 | 0.68 | 0.51 | -2.32E-02 |
| Common bottlenose dolphin | 3.18E-03 | 1.76E-03 | 1.81 | 0.08 | 8.63E-02 |
| Dwarf sperm whale | 3.44E-05 | 3.48E-04 | 0.10 | 0.92 | -4.30E-02 |
|  |  |  |  |  |  |
| **Proportion of all strandings in NEUS ~ SST anomaly (α′ = 8.51E-03)** | | | |  |  |
| Atlantic white-sided dolphin | *-2.91E-02* | *1.23E-02* | *-2.37* | *2.68E-02* | *0.16* |
| Long-finned pilot whale | **-1.37E-02** | **3.83E-03** | **-3.58** | **1.60E-03** | **0.33** |
| Harbor porpoise | **-8.81E-02** | **2.77E-02** | **-3.18** | **4.20E-03** | **0.27** |
| Common dolphin | **1.19E-01** | **2.25E-02** | **5.26** | **2.43E-05** | **0.53** |
| Common bottlenose dolphin | 2.43E-02 | 2.81E-02 | 0.87 | 0.40 | -1.06E-02 |
| Dwarf sperm whale | 1.32E-03 | 6.52E-04 | 2.02 | 0.05 | 1.14E-01 |
|  |  |  |  |  |  |
| **Proportion of all strandings in SEUS ~ SST anomaly (α′ = 8.51E-03)** | | | |  |  |
| Atlantic white-sided dolphin | -3.43E-03 | 2.60E-03 | -1.32 | 0.20 | 3.00E-02 |
| Long-finned pilot whale | 1.30E-03 | 1.33E-03 | 0.98 | 0.34 | -1.51E-03 |
| Harbor porpoise | 4.11E-03 | 2.14E-02 | 0.19 | 0.85 | -4.18E-02 |
| Common dolphin | -8.44E-03 | 6.43E-03 | -1.31 | 0.20 | 2.93E-02 |
| Common bottlenose dolphin | -6.78E-03 | 3.42E-02 | -0.20 | 0.84 | -4.17E-02 |
| Dwarf sperm whale | 8.30E-03 | 6.10E-03 | 1.36 | 0.19 | 3.41E-02 |
|  |  |  |  |  |  |
| **Change in human population ~ poleward distance (α′ = 7.30E-03)** | | | |  |  |
| Spatial range of analysis: |  |  |  |  |  |
| East coast of US | **-1.70E-02** | **2.74E-03** | **-6.21** | **5.89E-09** | **2.14E-01** |
| Poleward range of Atlantic white-sided  dolphin shift | -5.38E-05 | 5.39E-05 | -1.00 | 0.32 | -7.06E-05 |
| Poleward range of long-finned pilot whale  shift | **-1.38E-04** | **4.69E-05** | **-2.93** | **4.09E-03** | **6.47E-02** |
| Poleward range of harbor porpoise shift | -3.40E-05 | 5.23E-05 | -0.65 | 0.52 | -5.86E-03 |
| Poleward range of common dolphin shift | -5.86E-05 | 6.68E-05 | -0.88 | 3.82E-01 | -2.53E-03 |
| Poleward range of bottlenose dolphin shift | **-2.66E-04** | **5.57E-05** | **-4.78** | **6.81E-06** | **1.92E-01** |
| Poleward range of dwarf sperm whale shift | **-2.11E-04** | **3.48E-05** | **-6.06** | **1.61E-08** | **2.28E-01** |
